# Supplementary material for: Small RNA and mRNA Sequencing Reveal the Roles of microRNAs Involved in Pomegranate Female Sterility
Source: Int J Mol Sci. 2020 Jan 15;21(2):558. doi: 10.3390/ijms21020558 (PMC7013964; doi:10.3390/ijms21020558)
Supplement: Supplementary file 1 [file ijms-21-00558-s001.pdf]

Supplementary Materials

Small RNA and mRNA Sequencing Reveal Roles of microRNAs Involved in Pomegranate Female Sterility

Lina Chen, Xiang Luo, Xuanwen Yang, Dan Jing, Xiaocong Xia, Haoxian Li, Krishna Poudel and Shangyin Cao *

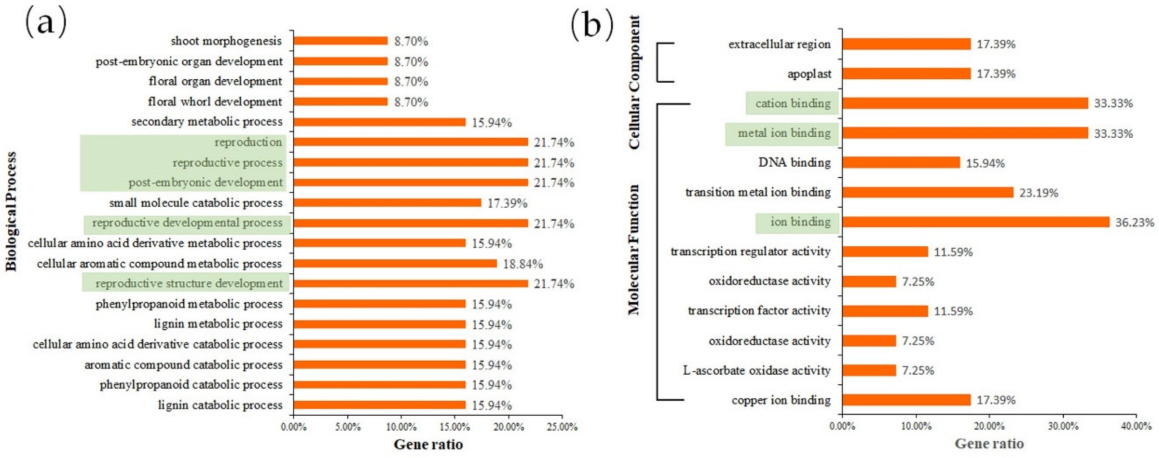

Figure S1. Gene ontology-based term classification of different expressed miRNA targets. (A) Biological Process, (B) Molecular Function and Cellular Component.

Table S1 Reads statistics of 18 small RNA libraries

Sample Name	Clean Reads	Percent of mapping to genome (%)	GC (%)	Unique sRNAs	Percent of mapping to genome (%)
ATNSI-1	23,066,631	87.60%	47.49%	9,603,258	81.18%
ATNSI-2	22,602,684	87.43%	48.79%	9,845,088	80.11%
ATNSI-3	22,894,680	87.15%	49.60%	8,896,708	79.82%
ATNSII-1	22,323,902	88.59%	49.36%	9,123,153	81.41%
ATNSII-2	22,691,179	83.96%	47.06%	9,589,434	77%
ATNSII-3	22,616,195	88.40%	49.81%	8,687,435	80.81%
ATNSIII-1	22,952,314	89.62%	50.09%	8,408,662	82.21%
ATNSIII-2	22,891,373	88.70%	49.84%	8,710,080	81.48%
ATNSIII-3	23,020,059	88.78%	48.37%	8,884,118	80.69%
TNSI-1	22,809,902	86.73%	48.56%	9,518,020	79.49%
TNSI-2	22,291,141	86.45%	49.59%	9,556,047	79.46%
TNSI-3	22,676,996	86.79%	49.42%	9,448,670	79.95%
TNSII-1	22,615,609	88.79%	49.46%	8,671,187	81.32%
TNSII-2	22,793,881	88.32%	49.32%	9,059,022	80.34%
TNSII-3	22,917,507	88.43%	49.39%	8,699,013	80.18%
TNSIII-1	23,009,715	88.95%	48.52%	9,230,450	81.50%
TNSIII-2	22,688,371	84.82%	50.01%	9,116,140	78%
TNSIII-3	22,897,914	88.21%	48.00%	10,245,633	81.40%

Table S2 Identification of miRNAs in female sterile and female fertile pistils

Note：the average value of each miRNA's reads counts. The novel miRNAs were highlighted in bold.

miR_name	Sequence	Length	ATNSI	ATNSII	ATNSIII	TNSI	TNSII	TNSIII
Pg-miR4376	TGCAGGAGGGATGACGCCGTC	21	259.5	1523.33	3283	411	994	1006.33
Pg-miR171b	TGATTGAGCCGTGCCAATATC	21	12029.5	13645.33	9562	15932	14717	13618.33
Pg-miR171b-3p	TTGAGCCGTGCCAATATCGCG	21	44974	29881.67	28245.5	31235.67	35787.33	27788.67
Pg-miR170-5p	TATTGGCCTGGTTCACTCAGA	21	7590.5	11542	7182.5	6509	9848	13547.67
Pg-miR399b	TGCCAAAGGAGAGTTGCCCTC	21	1411.5	2304.33	1544.5	1073	3249.67	2192
Pg-miR393a-3p	GATCATGCTATCCCTTTGGAT	21	2451	2527	1988.5	1502.67	2003.67	1803.67
Pg-miR166a-3p	TCGGACCAGGCTTCATTCCCC	21	117796	168465.7	225498.5	121906	129051	132769.3
Pg-miR6478	CCGACCTTAGCTCAGTTGGTA	21	3468	8175	9713.5	5616.33	6720.33	7667.33
Pg-miR167f-3p	AGATCATGTGGTAGCTTCACC	21	110	169.67	73.5	101	296.67	332.67
Pg-miR167h	TGAAGCTGCCAGCATGATCTTA	22	5212.5	10977.67	10050.5	8374.67	14113.33	35299
Pg-miR3627b	ATGTCGCAGGAGAGATGGCACG	22	6.5	22	33	3.67	165	0
Pg-miR8692	AACGGTCGTGCTGACGTGGCACA	23	3150	2991.67	3584	3242	3295.33	3212.33
Pg-miR160a-3p	GCGTATGAGGAGCCATGCATA	21	2572.5	878.33	660.5	2826	1696	983.33
Pg-miR160a-5p	TGCCTGGCTCCCTGTATGCCA	21	67097.5	63764.67	46611.5	29813.67	44138	29123
Pg-miR408-3p	ATGCACTGCCTCTTCCCTGGTC	22	736	5509.33	1303.5	606	742.67	760
Pg-miR479	CGTGATGTTGGTTCGGCTCATC	22	486.5	1518	1820	70.67	1852.67	2107.67
Pg-miR2111g-3p	AGCCTCGGGATTGCGGATTACC	22	135.5	69	60.5	80	105	101.67
Pg-miR169r-3p	GGCAGGTTGTCCTTGGCTACA	21	267	191.33	447	218.67	106.67	134.67
Pg-miR390d-3p	CGCTATCCATCCTGAGTTTTA	21	919.5	478.33	415.5	1362	557	633.67
Pg-miR2916	TGGGGGCTCGAAGACGATCAGA	22	2120.5	2992.33	3241.5	2528.33	3369	2843.67
Pg-miR6300	GTCGTTGTAGTATAGTGGTG	20	154.5	408.67	365.5	1304	137.33	447.33
Pg-miR164a	TGGAGAAGCAGGGCACGTGCA	21	361.5	2115	708.5	809.67	613.33	740
Pg-miR164g-3p	CACGTGCTCCCCTTCTCCAAC	21	28	20	12	21.33	18	17.33

Pg-miR397a	TCATTGAGTGCAGCGTTGATG	21	25.5	977.67	26.5	31	22.33	40
Pg-miR395a	CTGAAGTGTTTGGGGGAACTC	21	352.5	856.67	260.5	404.67	620.67	1364.33
Pg-miR398b	TGTGTTCTCAGGTCGCCCCTG	21	2195	28322.33	65872.5	2386.33	5684.67	3940.33
Pg-miR1046-3p	TGTGAAAAATCTGAAAAGTTC	21	48.5	33	27	53	46.33	44
Pg-miR398a-3p	TGTGTTTTCAGGTCACCCCTT	21	242.5	579.67	8704.5	347.33	1045.67	520.67
Pg-miR393h	TTCCAAAGGGATCGCATTGATC	22	72.5	351.67	644.5	162	352.67	311
Pg-miR169b-5p	CAGCCAAGGATGACTTGCCGG	21	1044	558	657.5	995.67	600.33	713.67
Pg-miR396b-5p	TTCCACAGCTTTCTTGAACTT	21	4768.5	7985	9820.5	3395.67	7799.67	11696
Pg-miR1077-5p	TTCAATGGTCCGATGCGTGGC	21	428	1220	1409.5	268	947.33	0
Pg-miR168a-3p	CCCGCCTTGCATCAACTGAAT	21	12236.5	11138	10903.5	13139.67	15976.67	17546.67
Pg-miR168a-5p	TCGCTTGGTGCAGGTCGGGAA	21	61008	63610.33	58785	58291.33	70168.33	71556.33
Pg-miR394a	TTGGCATTCTGTCCACCTCC	20	330.5	264.33	408	187.33	286.67	288.33
Pg-miR482-5p	GGAATGGGCGACTTGGGAGTA	21	5227.5	3323.33	1823.5	6515.67	5562.33	6298.33
Pg-miR482a-3p	TTCCCAAGGCCGCCCATTCCGA	22	1975.5	3441.33	3902.5	1782.33	3033.33	5314
Pg-miR9496	CTTGGTTGGGCTTGATGACTCC	22	454.5	852.67	826	683.67	926.33	715.67
Pg-miR156c	TTGACAGAAGAGAGAGAGCAC	21	155	609.33	304.5	44.33	710	1467.33
Pg-miR390a-5p	AAGCTCAGGAGGGATAGCGCC	21	3899	2577.33	2084	4143.67	2974.33	2313
Pg-miR162a-3p	TCGATAAACCTCTGCATCCAG	21	2806	3888	3623.5	2401.67	3376.67	3621
Pg-miR157a-5p	TTGACAGAAGATAGAGAGCAC	21	138	136	131	238.67	232.33	394.67
Pg-miR157d-3p	GCTCTCTATGCTTCTGTCATC	21	160	157	101.5	162.67	214.67	293.67
Pg-miR159a	TTTGGATTGAAGGGAGCTCTA	21	89466	87233.67	67640	93130	77461.33	75606.67
Pg-miR535a	TGACAGCGAGAGAGAGCACGC	21	378	1098	433.5	545.33	524.33	531
Pg-miR1078	TTATGGATGTTCAATTTTGTCGAT	24	181.5	163.67	158.5	109	129.67	175
Pg-miR1172.1	AGAGATTGCAAGAAGCACGGGCGC	24	992	262.67	0	1781.67	323.67	187.67
Pg-miR1425-3p	GCTAGCAAGACTCGTGATCTTAAT	24	29.5	25	33	23.33	27.33	22.67
Pg-miR1511-3p	AACCTGCTCTGATACCATGAAAGT	24	445.5	405.67	416	380.33	293.67	577.67
Pg-miR1514a-3p	ATGCCTCGTTTTATAATGAAATA	23	0	533.33	254.5	410.67	0	596.67

Pg-miR163-5p.1	CGGTTCCAGCTCATCTTCAGA	21	998	1249	1123	950.33	1147.67	937
Pg-miR165a-3p	TCGGACCAGGCTTCATCCCCC	21	52	107.67	117.5	60.33	31.33	96.33
Pg-miR2199	TGATAACTTGACGGATCGC	19	6518	6578.67	9939	6332	9338	6059.67
Pg-miR2592s-3p	AAATGCTGAGCTCGGATGTCTGTT	24	0	38.67	0	70.33	46.33	35.67
Pg-miR2627	TTTCGGTACTCTAACTTGCTAGGC	24	415.5	579.67	608.5	478	622.33	741.67
Pg-miR2644	CAACTTCAGATCATTGTGTGT	21	383.5	394.33	349	335.33	369	325.67
Pg-miR2863a	TTGTCCCATTGGCTGACGTTTAGC	24	209	158.67	64.5	270.67	158.33	156.67
Pg-miR319a-3p	TTGGACTGAAGGGAGCTCCCT	21	61133	18770	14125	77872	21695	8038.67
Pg-miR3440b-3p	TGTGATTGGTACAAGTGGAAGGTA	24	42.5	55.33	56.5	48.33	58.67	63.33
Pg-miR403c-5p	TTTGTGCGTGTCTGAGCCTC	20	32.5	14	53.5	31	0	0
Pg-miR4240	TGACAAGACCCGTAACAAGTTATC	24	1207	955.67	368	1596.33	916	1091
Pg-miR4387a	AACAGACGATGACGTGTATCACT	23	347	434	573	422	470	595.33
Pg-miR4414b	TGTGAATGATGCGGGAGATAC	21	117	43	66.5	96.67	133.33	69
Pg-miR444b.1	TGTTGTCTCAAGCTTGCTGCC	21	0	2810	0	15	7.33	55.33
Pg-miR447a-3p	TTGGGAAACGAGTATTTTTGTTGT	24	4643.5	4699.33	4646.5	6321.67	4181.33	4924.33
Pg-miR5054	CGTGCCCCACGGTGGGCGCCA	21	37.5	6160.67	17741.5	103.67	6596	35.67
Pg-miR5059	CGATCCTGGGCAGCAACACCA	21	197	212.67	255.5	307	254.33	112.67
Pg-miR5077	TTCACGTCGGGTTCACCA	18	1197	842	814.5	435.67	911	324
Pg-miR5152-3p	AGTCCTGCTATACCCACCA	19	68.5	107	159.5	76.33	91	0
Pg-miR5223	CGGGATTTATACTTGAAGCTGC	22	83.5	84.33	0	106.67	110.33	98
Pg-miR528-5p	TGGAAGGGGCATGCAGAGGAG	21	0	5545	0	20.33	7.67	120
Pg-miR5570	AAGAGAAAGTCAGACATGTCA	21	115	114.67	86.5	147	112.67	116.33
Pg-miR5653	GAGTTGAGTTGAGTTGAGTTGAGT	24	747.5	603.33	681	357	515.33	536.33
Pg-miR5663-5p	CGCCATAGGATTTCATTCTCA	21	72	150.67	70.5	60	114	58
Pg-miR5671a	CATGGTGGTGACGGGTGAC	19	0	819.33	341.5	945	0	221.33
Pg-miR5813	AACAGCCTCTGGTCGATGGA	20	2438	3297.33	5779	3007	3682.33	3291
Pg-miR6105b	CACGCAAAAAGTACTTGGTCTCAA	24	0	0	20.5	22.33	6.67	21.67

Pg-miR6161a	GCTGACCGTATCCTTTTGCTGAA	23	0	0	0	87	164	0
Pg-miR6173	CTAGCCGTAAACGATGGATACT	22	25.5	69.33	51.5	47.33	47	52
Pg-miR6183	AGAGACCGAGCTTGGCTGCAAGTT	24	284	291	316.5	350.33	320.67	552
Pg-miR6231-3p	AGTATTTGTGTACTCATGGACAGT	24	23.5	29.67	20.5	20.67	26.67	35.67
Pg-miR6462e	TCTTCATGCTGTTTTTGTCTAA	22	376.5	509	461	357.67	377	345
Pg-miR7532a	GAACAGCCTCTGGTCGATGGA	21	1850.5	2193	4162.5	2297.67	2596.33	2299.33
Pg-miR7533a	GATCGGGGAGGAGTAGAAGCTGCG	24	0	0	0	80.33	0	0
Pg-miR7717a-5p	TCTTGGCATTCGTGATAAATTCAA	24	0	0	0	24	0	4.67
Pg-miR7782-3p	ACCTAGACTCTGATACCATATTGA	24	108	304.33	401.5	209	335.67	280
Pg-miR779.2	TAGATTGAGAACATTTCGTGATCT	24	71.5	82.33	81.5	72.67	49.33	93
Pg-miR8148	TAACGGTCGTGCTGACGTGGCATG	24	5745.5	7748.33	6109.5	7807.33	6940.33	6597.33
Pg-miR8155	AACCCGCTCTGATATCATGTAAAA	24	2856	219.67	207.5	245.33	246	284.33
Pg-miR8175	CGTTCCCCGGCAACGGCGCCA	21	3820	3830.67	6902	3686.67	4049.67	2640
Pg-miR827-5p	TTTGTTGATGGTCATTTAGTC	21	970.5	598.67	573.5	1241.33	320.67	439.33
Pg-miR842-5p	GACATGATGGATCCGACCAGATGG	24	0	0	0	0	12.67	34.67
Pg-miR858b	TTCGTTGTCTGTTCGACCTTG	21	1777821	1145384	1545759.5	908644.7	2154559	1141311
Pg-miR8609	ACTAAGATCCGAGAGCCTAAGCTC	24	32	30.33	23	32.33	40	34.67
Pg-miR862-3p	ATATGCTAGGATCTGACAATGAAG	24	3	1.67	9.5	2.67	4.67	0
Pg-miR8700	AGGACGAGACGGTTAACTGTGATT	24	7	2.67	0	10.67	11.67	12.33
Pg-miR8712	ATATCTTGTAGATGATCGTCTT	22	179	56.33	170	0	101.67	140.33
Pg-miR8723a	ATCTAACGAGTCAGCTGACGTGAC	24	283.5	71.67	115.5	224.33	269.33	246.67
Pg-miR894	GTTTCACGTCGGGTTCACCA	20	2849	3488.67	3501	1665.33	2383.33	1650
Pg-miR902j-5p	AATGTTGCCGCTTCTTCAT	19	0	1287	2757.5	0	2216.33	0
Pg-miR952b	AACGAGGATCCATTGGAG	18	646	2065.67	4508	2324.67	2329.67	0
Pg-miR9726	TGTAGGCATTATTTTTTTTT	21	107.5	90.33	49.5	64	80.33	58
Pg-miR9761	TCGAAGGCAAGGATATTTATGT	22	62	144	73	93.67	116.67	64.33
**Pg-miRN01**	TTCGTTGTCTGTTCGACCTGA	21	8860.5	1383.3	5999.5	3159.3	10952.7	1902.0

**Pg-miRN02**	GACTGCCGACTCATTCATTCA	21	5066.0	2437.0	3886.5	2252.3	4402.3	2917.7
**Pg-miRN03**	TTCCACAGCTTTCTTGAACTT	21	4535.0	2694.0	9187.0	3262.7	4437.0	7848.0
**Pg-miRN04**	AATTTCATCAGGTATCCAGAGT	22	6413.0	6273.7	11166.0	2950.7	2195.3	2360.7
**Pg-miRN05**	TCGATAAACCTCTGCATCCAG	21	2715.0	1823.3	3498.0	2306.7	1120.0	1438.3
**Pg-miRN06**	TAGCTGCCGACTCATTCATCCA	22	2757.5	1634.0	0.0	2271.3	1206.0	427.3
**Pg-miRN07**	TCGGACCAGGCTTCGTTCCTC	21	3048.0	0.0	3239.0	1739.3	1839.3	1245.7
**Pg-miRN08**	GATCATGCTATCCCTTTGGAT	21	2408.5	1246.3	985.5	1470.7	1594.7	1066.3
**Pg-miRN09**	GCGTATGAGGAGCCATGCATA	21	2481.5	538.0	0.0	2201.7	0.0	0.0
**Pg-miRN10**	TTCGGACCAGGCTTCATTCCCC	22	3560.0	2024.3	2351.0	2781.0	2193.3	975.7
**Pg-miRN11**	TTGAGCCGCGCCAATATCACTT	22	2607.0	0.0	214.0	1304.0	1747.7	244.0
**Pg-miRN12**	GGAGTCCTTGTTGGAGAGTGG	21	2054.0	337.3	857.5	360.3	2001.0	171.0
**Pg-miRN13**	GGAATGTTGTCTGGCTCGAGG	21	3407.0	1082.3	0.0	1254.7	1822.3	1172.0
**Pg-miRN14**	CCAAGTGATGATTGACGAGTTC	22	1866.0	2404.0	3218.5	1447.3	2412.3	697.7
**Pg-miRN15**	AAGCTCAGGAGGGATAGCGCC	21	2429.0	1407.0	919.0	1876.0	1049.7	974.7
**Pg-miRN16**	TCAAGTGATGATTGACGAGATC	22	1018.0	977.3	818.5	780.0	942.7	556.0
**Pg-miRN17**	GTTCAATAAAGCTGTGGGAAG	21	779.5	1195.0	1580.0	293.7	1183.3	1196.3
**Pg-miRN18**	ATTGGACTGAAGGGAGCTCCT	21	990.0	47.3	0.0	1555.7	0.0	0.0
**Pg-miRN19**	CAGCCAAGGATGACTTGCCGG	21	678.0	306.0	206.5	564.7	398.0	556.7
**Pg-miRN20**	TTTGTTGATGGTCATTTAGTC	21	927.5	563.0	528.0	260.0	155.7	375.3
**Pg-miRN21**	TGCCAAAGGAGAGTTGCCCTC	21	1067.5	1343.7	1041.0	920.3	1575.7	1849.7
**Pg-miRN22**	TTCAGACGAAACTGCTTATCCC	22	532.5	449.0	72.5	608.3	278.0	213.7
**Pg-miRN23**	GTGCTCTCTTTCGTTGTCATA	21	495.5	217.3	420.0	454.7	459.7	557.3
**Pg-miRN24**	CTTGGACCAGGCTTCATTCCC	21	362.0	392.0	804.5	165.7	542.0	412.7
**Pg-miRN25**	TTTCTACGGATCCTATCACGT	21	275.0	311.7	276.0	134.0	424.0	62.3
**Pg-miRN26**	CTCCGAGGCCCATGGGTAGGT	21	185.0	76.7	214.0	69.3	218.3	241.7
**Pg-miRN27**	TGCCAAAGGAGAGTTGCCCTG	21	417.5	428.7	545.0	177.3	384.3	121.3
**Pg-miRN28**	CTGAAGTGTTTGGGGGAACTC	21	316.5	663.0	148.0	197.7	360.7	1069.7

**Pg-miRN29**	ATTTGTTGATAGTTATCTTTT	21	171.5	39.7	10.5	96.0	53.7	19.7
**Pg-miRN30**	GTGGAGGGTTCGGGGTCGCTG	21	162.0	0.0	0.0	67.0	287.3	138.7
**Pg-miRN31**	TTGACAGAAGATAGAGAGCAC	21	136.0	136.7	72.5	213.7	0.0	279.7
**Pg-miRN32**	TTGCGATCCGCGTCCCTCGAT	21	102.5	79.3	103.5	76.7	99.0	30.3
**Pg-miRN33**	TTGACAGAAGAGAGAGAGCAC	21	87.5	211.3	234.0	36.0	623.7	791.0
**Pg-miRN34**	ACACTCGAGCTGAATTACTTA	21	98.5	51.7	87.5	96.3	97.0	84.7
**Pg-miRN35**	CCAAGTGATGATTGACAAGTTC	22	110.5	236.3	474.0	72.3	203.7	280.3
**Pg-miRN36**	TGAGCCGAATCAATATCACTC	21	58.0	0.0	0.0	13.0	119.7	250.7
**Pg-miRN37**	CGCTATCCATCCTGAGTTTTA	21	461.0	270.3	348.0	1187.3	462.0	346.0
**Pg-miRN38**	TTCCAAAGGGATCGCATTGATC	22	80.5	200.3	416.0	72.0	185.3	213.3
**Pg-miRN39**	GTGCGGGTCCAACTGCAGACG	21	51.0	154.3	190.5	36.7	44.7	78.0
**Pg-miRN40**	TGACAGAAGAGAGTGAGCACA	21	63.5	54.0	64.0	72.7	63.3	29.3
**Pg-miRN41**	AGCCTCGGGATTGCGGATTACC	22	105.5	70.3	15.0	76.3	101.7	43.3
**Pg-miRN42**	TACTAGCTGTAGGGATATTGC	21	36.0	52.7	33.5	58.3	55.3	53.3
**Pg-miRN43**	TTAGATTGCGGGTTATGGAAG	21	28.0	22.3	9.5	17.3	23.0	29.3
**Pg-miRN44**	TGCCAAAGGAGATTTGCCCGG	21	19.0	6.0	0.0	25.0	47.3	85.0
**Pg-miRN45**	TCATTGAGTGCAGCGTTGATG	21	26.5	10.3	11.5	19.0	20.7	18.7
**Pg-miRN46**	GACCTTGACCATTTCTTTTAG	21	32.5	65.3	16.5	46.3	46.3	25.7
**Pg-miRN47**	ATATTCTAAGCAGTGCTGACC	21	17.5	10.0	11.5	16.0	23.7	24.7
**Pg-miRN48**	TGTCTGGGTAATGCTGTTGAG	21	20.5	34.3	0.0	26.3	9.7	33.0
**Pg-miRN49**	TGGTACCATCGACTTTATAAT	21	12.0	13.3	3.0	7.3	5.0	9.7
**Pg-miRN50**	AAGCACAATGACTTCGATCGA	21	14.0	12.3	3.0	11.7	11.0	18.3
**Pg-miRN51**	AATATGTCAAATATATGCTTG	21	9.5	13.7	13.5	10.0	8.7	18.0
**Pg-miRN52**	AAACTCAGGATGGATAGCGCC	21	7.0	9.7	8.0	5.3	9.7	9.7
**Pg-miRN53**	TGAAGCTGCCAGCATGATCTGA	22	0.0	2411.0	2939.0	2508.0	1748.0	2011.7
**Pg-miRN54**	TATTCTGGTGTCCTAGGCGTAG	22	0.0	1900.7	3337.5	666.0	567.7	2305.3
**Pg-miRN55**	GCTGACTCTGATACCATGATAGA	23	0.0	1022.0	2383.5	1458.3	1453.0	0.0

**Pg-miRN56**	TGGAGAAGCAGGGCACGTGCA	21	0.0	2017.7	274.0	517.3	605.3	514.7
**Pg-miRN57**	GGCTGTACCCTAATAAGTGA	20	0.0	22.3	0.0	72.3	0.0	93.7
**Pg-miRN58**	CTCGGAAGTCGATTTTGCTCG	21	0.0	6.7	17.0	3.7	7.3	12.7

Table S3 Identification of common differentially expressed miRNAs between BFs and FMFs' pistils

miR-name	log2FoldChange (ATNSI/TNSI)	padj[a]	Significance	log2FoldChange (ATNSII/TNSII)	padj[a]	Significance	log2FoldChange (ATNSIII/TNSIII)	padj[a]	Significance
Pg-miR4376	-0.70	0.88		0.66	0.45		1.66	0.00	**
Pg-miR1077-5p	NA[b]	NA[b]		0.42	0.99		12.86	0.00	**
Pg-miR902j-5p	NA[b]	NA[b]		-0.72	0.99		13.82	0.00	**
Pg-miR952b	-1.85	1.00		-0.09	0.99		14.53	0.00	**
Pg-miR167f-3p	0.10	1.00		-0.75	0.26		-2.23	0.00	**
Pg-miR398b	-0.15	1.00		2.42	0.03	**	4.01	0.00	**
Pg-miR166a-3p	-0.06	1.00		0.45	0.37		0.72	0.00	**
Pg-miR5152-3p	-0.07	1.00		0.29	0.99		9.71	0.00	**
Pg-miR1172.1	-0.83	0.58		-0.24	0.99		-10.04	0.00	**
Pg-miR5223	-0.43	0.87		-0.32	0.37		-9.10	0.00	**
Pg-miR403c-5p	0.09	1.00		6.29	0.49		8.14	0.00	**
Pg-miR156c	1.94	1.00		-0.17	0.99		-2.32	0.00	**
Pg-miR842-5p	NA[b]	NA[b]		-6.04	0.54		-7.61	0.00	**
Pg-miR171b	-0.44	0.69		-0.05	0.99		-0.56	0.00	**
Pg-miR482-5p	-0.31	1.00		-0.72	0.99		-1.84	0.00	**
Pg-miR3627b	0.64	1.00		-2.83	0.93		7.44	0.00	**
Pg-miRN54	-11.94	0.04	*	1.82	0.99		0.49	0.00	
Pg-miR528-5p	-6.91	0.35		9.60	0.02	*	-9.37	0.00	**
Pg-miR170-5p	0.15	1.00		0.32	0.99		-0.97	0.00	**
Pg-miR4240	-0.47	0.87		0.12	0.99		-1.61	0.00	**
Pg-miRN48	-0.36	1.00		1.96	0.99		-7.52	0.00	**

Pg-miR444b.1	-6.52	0.42		8.69	0.05	*	-8.26	0.00	**
Pg-miRN44	-0.49	1.00		-2.99	0.63		-8.87	0.00	**
Pg-miR398a-3p	-0.62	1.00		-0.78	0.99		4.00	0.00	**
Pg-miR167h	-0.69	1.00		-0.34	0.99		-1.86	0.00	**
Pg-miR393h	-1.09	1.00		0.06	0.99		1.00	0.00	**
Pg-miR8700	-0.90	1.00		-2.08	0.86		-6.10	0.00	**
Pg-miRN13	1.52	0.36		-0.69	0.99		-12.69	0.02	*
Pg-miR5671a	-12.30	0.00	**	12.18	0.00	**	0.53	0.95	
Pg-miRN07	0.64	1.00		-13.21	0.00	**	1.35	0.84	
Pg-miRN30	1.12	1.00		-10.55	0.00	**	-9.58	0.10	
Pg-miR6161a	-9.07	0.15		-9.73	0.00	**	NA[b]	NA[b]	
Pg-miRN31	-0.77	0.78		9.60	0.00	**	-2.02	0.84	
Pg-miR6300	-2.89	0.35		1.65	0.00	**	-0.33	0.87	
Pg-miR8723a	0.25	1.00		-1.82	0.01	**	-1.13	0.69	
Pg-miR397a	-0.35	1.00		5.62	0.01	**	-0.63	0.74	
Pg-miRN11	0.90	1.00		-13.15	0.01	**	-0.28	0.98	
Pg-miRN22	-0.33	1.00		0.77	0.01	**	-1.60	0.82	
Pg-miR4414b	0.26	1.00		-1.58	0.01	*	-0.11	0.93	
Pg-miR408-3p	0.26	1.00		3.02	0.02	*	0.73	0.30	
Pg-miR165a-3p	-0.16	1.00		1.86	0.02	*	0.25	0.92	
Pg-miR827-5p	-0.37	1.00		0.98	0.02	*	0.34	0.37	
Pg-miRN09	-0.02	1.00		11.57	0.02	*	NA[b]	NA[b]	
Pg-miR160a-3p	-0.26	1.00		-0.90	0.03	*	-0.63	0.25	
Pg-miR858b	0.92	0.00	**	-0.86	0.03	*	0.38	0.69	
Pg-miR1514a-3p	-11.17	0.00	**	NA[b]	NA[b]		-1.27	0.84	
Pg-miR8712	9.89	0.00	**	-0.83	0.99		0.23	0.50	

Pg-miRN53	-26.84	0.00	**	0.50	0.99	0.47	0.93
Pg-miR7533a	-8.79	0.00	**	NA[b]	NA[b]	NA[b]	NA[b]
Pg-miRN01	1.43	0.00	**	-2.83	0.99	1.65	0.87
Pg-miR2592s-3p	-8.65	0.00	**	-0.26	0.99	-7.66	0.19
Pg-miRN57	-8.70	0.00	**	6.92	0.41	-9.01	0.12
Pg-miR6105b	-6.98	0.00	**	-5.13	0.68	-0.10	0.98
Pg-miR7717a-5p	-7.08	0.00	**	NA[b]	NA[b]	-4.67	0.62
Pg-miR7782-3p	-1.00	0.00	**	-0.07	0.99	0.47	0.86
Pg-miR390d-3p	-0.60	0.03	*	-0.16	0.99	-0.66	0.11
Pg-miRN56	-11.61	0.05	*	1.86	0.43	-0.97	0.92

Note: a: adjusted P-value

b: values were filtered：-0.5＜log2fold change＜0.5　and adjusted P-value ＞0.05

Table S4 Identification of common differentially expressed miRNAs among different stages of BFs

miR-name	log2FoldChange (TNSII/TNSI)	padj[a]	Significance	log2FoldChange (TNSIII/TNSI)	padj[a]	Significance	log2FoldChange (TNSIII/TNSII)	padj[a]	Significance
Pg-miRN18	-13.1796674	5.06E-20	**	-13.20032029	2.99E-20	**	NA[b]	NA[b]	
Pg-miR1514a-3p	-11.30191764	5.81E-19	**	0.343928281	0.466629		11.61318807	4.89E-20	**
Pg-miR5671a	-12.45706442	5.34E-16	**	-2.214342629	0.815249		Nab	NA[b]	
Pg-miRN01	1.602849408	5.34E-16	**	-30	1.94E-14	**	-2.668729388	0.96594	
Pg-miR858b	1.062078061	1.37E-14	**	0.135291341	0.947827		-0.946694677	0.052256	
Pg-miRN31	-10.41211783	3.31E-13	**	0.165305366	0.947827		10.53575631	0.068741	
Pg-miR7533a	-8.930407954	1.62E-11	**	-8.948753273	1.05E-11	**	NA[b]	NA[b]	
Pg-miRN57	-8.832779405	6.59E-11	**	0.128140766	0.947827		8.913342857	0.15272	
Pg-miR827-5p	-2.133474839	2.02E-08	**	-1.692569	7.53E-06	**	0.409095564	0.366129	
Pg-miR7717a-5p	-7.216149993	4.84E-07	**	-2.588967722	0.506935		4.522793767	0.797569	
Pg-miR1172.1	-2.605028893	1.55E-06	**	-3.405157079	1.74E-13	**	-0.821109835	0.210475	

Pg-miR479	4.457755564	1.39E-05	**	4.627874932	2.19E-10	**	0.149446515	0.987174	
Pg-miRN17	1.882261603	0.000176	**	1.874631626	0.000197	**	-0.032964261	0.987174	
Pg-miR396b-5p	1.000177521	0.000554	**	1.576229245	7.43E-12	**	0.541715391	0.068741	
Pg-miR167f-3p	1.384303016	0.000567	**	1.544281115	5.86E-08	**	0.135657065	0.987174	
Pg-miRN09	-13.82702684	0.003231	**	-13.83042936	0.002783	**	NA[b]	NA[b]	
Pg-miR390d-3p	-1.465793489	0.004716	**	-1.294085175	1.73E-08	**	0.15149157	0.987174	
Pg-miR902j-5p	13.33085434	0.004716	**	NA[b]	NA[b]		-13.55375035	0.008194	**
Pg-miR390a-5p	-0.67034416	0.008793	**	-1.050536064	3.84E-11	**	-0.409389107	0.15272	
Pg-miR4240	-0.992989433	0.011847	*	-0.752089585	0.122767		0.200282455	0.96594	
Pg-miRN37	-1.547860163	0.014978	*	-1.993703277	0.661745		-0.475352308	0.987174	
Pg-miR6300	-3.293951741	0.027715	*	-1.629861776	0.520026		1.670507688	0.071546	
Pg-miR8712	8.964562799	0.092877		9.371333835	1.13E-13	**	0.379100552	0.987174	
Pg-miR842-5p	5.943348141	0.41492		7.354899709	1.68E-08	**	1.41443468	0.96594	

Pg-miR170-5p	0.425415015	0.140215		0.864863615	1.47E-07	**	0.416220448	0.389804	
Pg-miR319a-3p	-1.976177273	0.103832		-3.419526998	2.31E-05	**	-1.475894757	0.242701	
Pg-miR160a-3p	-0.964969282	0.179919		-1.760476981	0.002029	**	-0.815992818	0.098213	
Pg-miRN38	1.195910555	0.064008		1.39425808	0.002288	**	0.172672106	0.987174	
Pg-miR952b	-0.104132797	0.98902		-13.72420962	0.002955	**	-13.62552347	0.008194	**
Pg-miRN55	-0.20168617	0.987489		-13.08320591	0.005194	**	NA[b]	NA[b]	
Pg-miR2627	0.178552556	0.983309		0.43061302	0.00656	**	0.227264915	0.9771	
Pg-miR393h	0.934599802	0.199722		0.751542087	0.008162	**	-0.210013676	0.987174	
Pg-miR167h	0.625672766	0.627739		1.933884499	0.009705	**	1.278248832	0.006047	**
Pg-miR171b	-0.303871043	0.310141		-0.42332186	0.013987		-0.146279397	0.855619	
Pg-miR6161a	0.625291036	0.983309		-9.179414033	0.083822		-9.84330834	3.37E-14	**
Pg-miR779.2	-0.797190883	0.377206		0.117819148	0.947827		0.872900831	0.006047	**
Pg-miR6183	-0.333830906	0.983309		0.435303924	0.698309		0.742882123	0.008194	**

Pg-miR1077-5p	1.596377939	0.983309		-24.58415789	3.85E-09	**	-12.32713481	0.02098	*
Pg-miR156c	3.98469873	0.377206		4.995378937	0.195954		0.994922649	0.02098	*

Note: a: adjusted P-value; b: values were filtered：-0.5＜log2fold change＜0.5 and adjusted P-value ＞0.05

Table S5 Identification of common differentially expressed miRNAs among different stages of FMFs

miR-name	log2FoldChange (ATNSII/ATNSI)	padj[a]	Significance	log2FoldChange (ATNSIII/ATNSI)	padj[a]	Significance	log2FoldChange (ATNSIII/ATNSII)	padj[a]	Significance
Pg-miRN07	-14.1	0.0	**	0.0	1.0		14.1	0.0	**
Pg-miRN11	-13.9	0.0	**	-3.7	0.5		NA[b]	NA[b]	
Pg-miR5671a	12.0	0.0	**	23.4	0.0	**	-1.3	1.0	
Pg-miR444b.1	26.4	0.0	**	NA[b]	NA[b]		NA[b]	NA[b]	
Pg-miR398b	3.6	0.0	**	4.8	0.0	**	1.2	0.1	
Pg-miRN30	-9.9	0.0	**	-9.9	0.0	**	NA[b]	NA[b]	
Pg-miRN56	13.3	0.0	**	NA[b]	NA[b]		-2.8	0.9	
Pg-miR4376	2.5	0.0	**	3.6	0.0	**	1.1	0.1	
Pg-miR528-5p	14.8	0.0	**	NA[b]	NA[b]		-14.8	0.0	**
Pg-miR156c	1.9	0.0	**	0.9	0.4		-1.0	0.0	*
Pg-miRN36	-8.4	0.0	**	-8.4	0.0	**	NA[b]	NA[b]	
Pg-miR169b-5p	-1.0	0.0	*	-0.8	0.1		0.3	0.9	
Pg-miR390d-3p	-1.0	0.0	*	-1.3	0.0	**	-0.2	1.0	
Pg-miR1172.1	-2.0	0.0	*	-12.5	0.0	**	-10.5	0.0	**
Pg-miRN53	13.6	0.0	*	13.9	0.0	**	0.3	1.0	
Pg-miR160a-3p	-1.6	0.0	*	-2.1	0.0	**	-0.4	1.0	
Pg-miR8723a	-2.1	0.0	*	-1.4	0.5		0.7	1.0	
Pg-miRN55	12.3	0.0	*	NA[b]	NA[b]		1.2	1.0	
Pg-miRN38	1.2	0.0	*	2.3	0.0	**	1.1	0.0	*
Pg-miRN54	NA[b]	NA[b]		14.0	0.0	**	0.9	1.0	
Pg-miR902j-5p	NA[b]	NA[b]		13.8	0.0	**	1.0	1.0	
Pg-miRN09	-2.3	0.9		-13.8	0.0	**	-11.4	0.1	
Pg-miRN13	-1.8	1.0		-14.3	0.0	**	-12.5	0.0	**

Pg-miRN18	-4.5	0.6	-12.5	0.0	**	NA[b]	NA[b]	
Pg-miRN06	-0.8	1.0	-14.0	0.0	**	-13.1	0.0	**
Pg-miR5223	-0.1	1.0	-8.9	0.0	**	-8.8	0.0	**
Pg-miR398a-3p	1.2	0.1	5.1	0.0	**	3.9	0.0	**
Pg-miR4240	-0.4	0.9	-1.8	0.0	**	-1.4	0.1	
Pg-miR166a-3p	0.4	0.5	0.8	0.0	**	0.4	0.5	
Pg-miRN35	1.0	0.2	2.0	0.0	**	1.0	0.3	
Pg-miRN48	0.7	1.0	-6.9	0.0	**	-21.0	0.0	**
Pg-miR319a-3p	-1.8	0.5	-2.2	0.0	**	-0.4	1.0	
Pg-miR6105b	NA[b]	NA[b]	6.7	0.0	**	6.8	0.0	**
Pg-miRN12	-2.7	1.0	-1.4	0.0	**	1.4	1.0	
Pg-miRN58	5.1	0.6	6.4	0.0	*	1.3	1.0	
Pg-miR165a-3p	1.0	0.5	1.1	0.0	*	0.2	1.0	
Pg-miR482-5p	-0.7	1.0	-1.6	0.0	*	-0.9	1.0	
Pg-miRN44	-1.7	1.0	-6.8	0.0	*	-5.0	0.9	

Note: a: adjusted P-value; b: values were filtered：-0.5＜log2fold change＜0.5　and adjusted P-value ＞0.05

Table S6 miRNA targets identified in pistils of BFs and FMFs

miRNA	Target	annotation
Pg-miR4376	*Gglean025376.1*	PREDICTED: uncharacterized protein LOC103696520 [*Phoenix dactylifera*]
Pg-miR4376	*Gglean014835.1*	PREDICTED: uncharacterized methyltransferase C3H7.11-like isoform X1 [*Eucalyptus grandis*]
Pg-miR171b	*Gglean025172.1*	PREDICTED: scarecrow-like protein 15 [*Eucalyptus grandis*]
Pg-miR171b	*Gglean006729.1*	PREDICTED: scarecrow-like protein 6 [*Eucalyptus grandis*]
Pg-miR171b-3p	*Gglean025172.1*	PREDICTED: scarecrow-like protein 15 [*Eucalyptus grandis*]
Pg-miR171b-3p	*Gglean006729.1*	PREDICTED: scarecrow-like protein 6 [*Eucalyptus grandis*]
Pg-miR399b	*Gglean019717.1*	laccase family protein [*Populus trichocarpa*]
Pg-miR399b	*Gglean026841.1*	PREDICTED: probable inorganic phosphate transporter 1-3 [*Eucalyptus grandis*]
Pg-miR166a-3p	*Gglean012177.1*	hypothetical protein JCGZ_09223 [*Jatropha curcas*]
Pg-miR166a-3p	*Gglean013966.1*	PREDICTED: homeobox-leucine zipper protein ATHB-8-like [*Eucalyptus grandis*]
Pg-miR166a-3p	*Gglean027183.1*	HB1-like protein [*Eucalyptus cladocalyx*]
Pg-miR166a-3p	*Gglean031286.1*	DNA binding protein, putative [*Ricinus communis*]
Pg-miR167h	*Gglean022780.1*	PREDICTED: probable phosphoribosylformylglycinamidine synthase, chloroplastic/mitochondrial [*Eucalyptus grandis*]
Pg-miR8692	*Gglean025175.1*	NA[a]
Pg-miR8692	*Gglean021649.1*	NA[a]
Pg-miR160a-5p	*Gglean024332.1*	hypothetical protein JCGZ_12025 [*Jatropha curcas*]
Pg-miR160a-5p	*Gglean003992.1*	PREDICTED: auxin response factor 22 isoform X1 [*Eucalyptus grandis*]
Pg-miR160a-5p	*Gglean026255.1*	PREDICTED: auxin response factor 18-like [*Eucalyptus grandis*]
Pg-miR160a-5p	*Gglean025700.1*	hypothetical protein CISIN_1g005075mg [*Citrus sinensis*]
Pg-miR408-3p	*Gglean029859.1*	Basic blue protein [*Medicago truncatula*]
Pg-miR408-3p	*Gglean030438.1*	Laccase 3 [*Theobroma cacao*]
Pg-miR408-3p	*Gglean009342.1*	hypothetical protein PRUPE_ppa021383mg [*Prunus persica*]
Pg-miR408-3p	*Gglean022110.1*	NA[a]
Pg-miR408-3p	*Gglean022081.1*	PREDICTED: zinc finger with UFM1-specific peptidase domain protein [*Eucalyptus grandis*]

Pg-miR408-3p	*Gglean027383.1*	PREDICTED: callose synthase 7 [*Prunus mume*]
Pg-miR408-3p	*Gglean010813.1*	PREDICTED: pentatricopeptide repeat-containing protein At3g58590 [*Eucalyptus grandis*]
Pg-miR408-3p	*Gglean009354.1*	Basic blue protein, putative [*Ricinus communis*]
Pg-miR169r-3p	*Gglean009790.1*	NA[a]
Pg-miR6300	*Gglean009658.1*	PREDICTED: probable polyamine transporter At1g31830 isoform X2 [*Eucalyptus grandis*]
Pg-miR6300	*Gglean008406.1*	PREDICTED: putative pentatricopeptide repeat-containing protein At5g08310, mitochondrial [*Eucalyptus grandis*]
Pg-miR6300	*Gglean022739.1*	PREDICTED: OBERON-like protein [*Populus euphratica*]
Pg-miR6300	*Gglean013458.1*	myb family transcription factor family protein [*Populus trichocarpa*]
Pg-miR6300	*Gglean016906.1*	PREDICTED: peroxisome biogenesis protein 7 [*Eucalyptus grandis*]
Pg-miR6300	*Gglean018041.1*	PREDICTED: regulatory protein NPR5 [*Vitis vinifera*]
Pg-miR164a	*Gglean022642.1*	hypothetical protein L484_021218 [*Morus notabilis*]
Pg-miR164a	*Gglean026964.1*	PREDICTED: UDP-glucuronic acid decarboxylase 6-like [*Populus euphratica*]
Pg-miR164a	*Gglean003381.1*	hypothetical protein PRUPE_ppa008801mg [*Prunus persica*]
Pg-miR164a	*Gglean003444.1*	PREDICTED: protein CUP-SHAPED COTYLEDON 2 [*Eucalyptus grandis*]
Pg-miR164a	*Gglean007834.1*	hypothetical protein PRUPE_ppa019065mg, partial [*Prunus persica*]
Pg-miR164g-3p	*Gglean017528.1*	OSJNBb0072N21.12 [*Oryza sativa* Japonica Group]
Pg-miR164g-3p	*Gglean022811.1*	Uncharacterized protein TCM_035725 [*Theobroma cacao*]
Pg-miR164g-3p	*Gglean025347.1*	hypothetical protein VITISV_044312 [*Vitis vinifera*]
Pg-miR397a	*Gglean026885.1*	LAC [*Punica granatum*]
Pg-miR397a	*Gglean030438.1*	Laccase 3 [*Theobroma cacao*]
Pg-miR397a	*Gglean021592.1*	PREDICTED: laccase-4-like [*Eucalyptus grandis*]
Pg-miR397a	*Gglean021664.1*	PREDICTED: laccase-17-like [*Eucalyptus grandis*]
Pg-miR397a	*Gglean019707.1*	PREDICTED: laccase-7-like [*Eucalyptus grandis*]
Pg-miR397a	*Gglean021685.1*	PREDICTED: laccase-7 [*Vitis vinifera*]
Pg-miR397a	*Gglean014191.1*	PREDICTED: laccase-17 [*Sesamum indicum*]
Pg-miR397a	*Gglean018490.1*	LAC [*Punica granatum*]
Pg-miR397a	*Gglean019717.1*	laccase family protein [*Populus trichocarpa*]

Pg-miR397a	*Gglean009496.1*	PREDICTED: bark storage protein A-like [*Vitis vinifera*]
Pg-miR397a	*Gglean021591.1*	Laccase/Diphenol oxidase family protein isoform 1 [*Theobroma cacao*]
Pg-miR397a	*Gglean019708.1*	PREDICTED: laccase-7 [*Eucalyptus grandis*]
Pg-miR395a	*Gglean006692.1*	NA[a]
Pg-miR395a	*Gglean022778.1*	PREDICTED: ATP sulfurylase 1, chloroplastic-like [*Eucalyptus grandis*]
Pg-miR395a	*Gglean015303.1*	Early nodulin 70 family protein [*Populus trichocarpa*]
Pg-miR398b	*Gglean005184.1*	PREDICTED: purple acid phosphatase 22-like isoform X1 [*Eucalyptus grandis*]
Pg-miR393h	*Gglean017109.1*	auxin signaling f-box 2 -like protein [*Gossypium arboreum*]
Pg-miR393h	*Gglean006519.1*	PREDICTED: protein TRANSPORT INHIBITOR RESPONSE 1 [*Vitis vinifera*]
Pg-miR169b-5p	*Gglean000395.1*	PREDICTED: cytochrome b5 [*Eucalyptus grandis*]
Pg-miR169b-5p	*Gglean009790.1*	NA[a]
Pg-miR169b-5p	*Gglean008635.1*	hypothetical protein EUGRSUZ_E03531 [*Eucalyptus grandis*]
Pg-miR396b-5p	*Gglean029554.1*	PREDICTED: putative B3 domain-containing protein At3g24850 [*Vitis vinifera*]
Pg-miR396b-5p	*Gglean002260.1*	PREDICTED: small glutamine-rich tetratricopeptide repeat-containing protein 2 [*Tarenaya hassleriana*]
Pg-miR396b-5p	*Gglean026508.1*	hypothetical protein JCGZ_06261 [*Jatropha curcas*]
Pg-miR396b-5p	*Gglean011692.1*	hypothetical protein EUGRSUZ_D00484 [*Eucalyptus grandis*]
Pg-miR396b-5p	*Gglean017067.1*	hypothetical protein PRUPE_ppa018570mg, partial [*Prunus persica*]
Pg-miR396b-5p	*Gglean012701.1*	PREDICTED: uncharacterized protein LOC104417748 [*Eucalyptus grandis*]
Pg-miR1077-5p	*Gglean016825.1*	F15O4.13 [*Arabidopsis thaliana*]
Pg-miR1077-5p	*Gglean021499.1*	unknown protein [*Oryza sativa* Japonica Group]
Pg-miR168a-5p	*Gglean028715.1*	PREDICTED: protein argonaute 1-like isoform X3 [*Nicotiana tomentosiformis*]
Pg-miR168a-5p	*Gglean028716.1*	PREDICTED: protein argonaute 1 [*Eucalyptus grandis*]
Pg-miR394a	*Gglean020297.1*	PREDICTED: F-box only protein 6 [*Eucalyptus grandis*]
Pg-miR482a-3p	*Gglean016618.1*	hypothetical protein EUGRSUZ_E00730, partial [*Eucalyptus grandis*]
Pg-miR482a-3p	*Gglean008494.1*	PREDICTED: polygalacturonase At1g48100-like [*Eucalyptus grandis*]
Pg-miR482a-3p	*Gglean030072.1*	PREDICTED: protein NSP-INTERACTING KINASE 1 [*Eucalyptus grandis*]
Pg-miR9496	*Gglean006494.1*	hypothetical protein POPTR_0014s12800g [*Populus trichocarpa*]

Pg-miR9496	*Gglean004507.1*	Movement protein [*Medicago truncatula*]
Pg-miR9496	*Gglean004532.1*	Movement protein [*Medicago truncatula*]
Pg-miR156c	*Gglean028306.1*	LIGULELESS1 protein, putative [*Theobroma cacao*]
Pg-miR156c	*Gglean016197.1*	PREDICTED: squamosa promoter-binding-like protein 16 [*Vitis vinifera*]
Pg-miR156c	*Gglean028488.1*	PREDICTED: squamosa promoter-binding-like protein 13A [*Vitis vinifera*]
Pg-miR156c	*Gglean000847.1*	LIGULELESS1 protein, putative [*Theobroma cacao*]
Pg-miR156c	*Gglean001912.1*	PREDICTED: squamosa promoter-binding-like protein 7 [*Eucalyptus grandis*]
Pg-miR156c	*Gglean016208.1*	PREDICTED: squamosa promoter-binding-like protein 2 [*Eucalyptus grandis*]
Pg-miR156c	*Gglean004741.1*	unnamed protein product [*Vitis vinifera*]
Pg-miR390a-5p	*Gglean012251.1*	Leucine-rich repeat receptor protein kinase EXS precursor, putative [*Ricinus communis*]
Pg-miR390a-5p	*Gglean025452.1*	PREDICTED: uncharacterized protein LOC101315171 [*Fragaria vesca* subsp. vesca]
Pg-miR390a-5p	*Gglean022261.1*	hypothetical protein EUGRSUZ_F02430 [*Eucalyptus grandis*]
Pg-miR390a-5p	*Gglean000925.1*	PREDICTED: dnaJ homolog subfamily B member 1 [*Eucalyptus grandis*]
Pg-miR390a-5p	*Gglean003665.1*	PREDICTED: serine/threonine-protein kinase BRI1-like 1 [*Populus euphratica*]
Pg-miR157a-5p	*Gglean016208.1*	PREDICTED: squamosa promoter-binding-like protein 2 [*Eucalyptus grandis*]
Pg-miR157a-5p	*Gglean000847.1*	LIGULELESS1 protein, putative [*Theobroma cacao*]
Pg-miR157a-5p	*Gglean000847.1*	LIGULELESS1 protein, putative [*Theobroma cacao*]
Pg-miR157a-5p	*Gglean004741.1*	unnamed protein product [*Vitis vinifera*]
Pg-miR157a-5p	*Gglean028488.1*	PREDICTED: squamosa promoter-binding-like protein 13A [*Vitis vinifera*]
Pg-miR157a-5p	*Gglean028306.1*	LIGULELESS1 protein, putative [*Theobroma cacao*]
Pg-miR157a-5p	*Gglean012943.1*	unnamed protein product [*Vitis vinifera*]
Pg-miR157d-3p	*Gglean010012.1*	PREDICTED: uncharacterized protein LOC104421582 [*Eucalyptus grandis*]
Pg-miR159a	*Gglean017814.1*	PREDICTED: transcription factor GAMYB [*Eucalyptus grandis*]
Pg-miR159a	*Gglean006069.1*	PREDICTED: uncharacterized protein LOC104450247 [*Eucalyptus grandis*]
Pg-miR159a	*Gglean006748.1*	PREDICTED: transcription factor MYB48 [*Vitis vinifera*]
Pg-miR159a	*Gglean030065.1*	PREDICTED: histone acetyltransferase HAC12-like [*Eucalyptus grandis*]
Pg-miR159a	*Gglean007062.1*	PREDICTED: uncharacterized protein LOC104227642 [*Nicotiana sylvestris*]

Pg-miR159a	*Gglean024643.1*	PREDICTED: uncharacterized protein LOC104887816 [*Beta vulgaris* subsp. vulgaris]
Pg-miR159a	*Gglean016054.1*	PREDICTED: transcription factor GAMYB [*Eucalyptus grandis*]
Pg-miR159a	*Gglean028556.1*	Protein FRIGIDA -like protein [*Gossypium arboreum*]
Pg-miR535a	*Gglean000349.1*	NA[a]
Pg-miR535a	*Gglean012602.1*	PREDICTED: monosaccharide-sensing protein 2-like [*Eucalyptus grandis*]
Pg-miR1511-3p	*Gglean027463.1*	PREDICTED: defensin-like protein 182 [*Malus domestica*]
Pg-miR163-5p.1	*Gglean026772.1*	PREDICTED: probable inactive serine/threonine-protein kinase bub1 [*Eucalyptus grandis*]
Pg-miR163-5p.1	*Gglean004426.1*	PREDICTED: probable acyl-activating enzyme 1, peroxisomal [*Eucalyptus grandis*]
Pg-miR163-5p.1	*Gglean009948.1*	Movement protein [*Medicago truncatula*]
Pg-miR163-5p.1	*Gglean004532.1*	Movement protein [*Medicago truncatula*]
Pg-miR163-5p.1	*Gglean004507.1*	Movement protein [*Medicago truncatula*]
Pg-miR163-5p.1	*Gglean008898.1*	PREDICTED: LOW QUALITY PROTEIN: leucine-rich repeat receptor-like tyrosine-protein kinase At2g41820 [*Eucalyptus grandis*]
Pg-miR163-5p.1	*Gglean007112.1*	putative polyprotein [*Nicotiana tabacum*]
Pg-miR165a-3p	*Gglean031286.1*	DNA binding protein, putative [*Ricinus communis*]
Pg-miR165a-3p	*Gglean027183.1*	HB1-like protein [*Eucalyptus cladocalyx*]
Pg-miR165a-3p	*Gglean013966.1*	PREDICTED: homeobox-leucine zipper protein ATHB-8-like [*Eucalyptus grandis*]
Pg-miR165a-3p	*Gglean012177.1*	hypothetical protein JCGZ_09223 [*Jatropha curcas*]
Pg-miR319a-3p	*Gglean024699.1*	NA[a]
Pg-miR319a-3p	*Gglean017814.1*	PREDICTED: transcription factor GAMYB [*Eucalyptus grandis*]
Pg-miR319a-3p	*Gglean016054.1*	PREDICTED: transcription factor GAMYB [*Eucalyptus grandis*]
Pg-miR319a-3p	*Gglean022391.1*	PREDICTED: kinesin-like protein KIF22-A [*Prunus mume*]
Pg-miR319a-3p	*Gglean028556.1*	Protein FRIGIDA -like protein [*Gossypium arboreum*]
Pg-miR319a-3p	*Gglean013014.1*	Uncharacterized protein F383_17343 [*Gossypium arboreum*]
Pg-miR319a-3p	*Gglean000711.1*	PREDICTED: A/G-specific adenine DNA glycosylase isoform X1 [*Eucalyptus grandis*]
Pg-miR444b.1	*Gglean003233.1*	PREDICTED: putative leucine-rich repeat-containing protein DDB_G0290503 [*Eucalyptus grandis*]
Pg-miR444b.1	*Gglean004566.1*	PREDICTED: MADS-box transcription factor 23-like isoform X1 [*Eucalyptus grandis*]
Pg-miR5054	*Gglean029036.1*	homeobox protein, putative [*Ricinus communis*]

Pg-miR5054	*Gglean022002.1*	PREDICTED: scarecrow-like protein 8 [*Eucalyptus grandis*]
Pg-miR5054	*Gglean013606.1*	NA[a]
Pg-miR5054	*Gglean020952.1*	PREDICTED: probable sarcosine oxidase [*Eucalyptus grandis*]
Pg-miR5077	*Gglean007189.1*	PREDICTED: pentatricopeptide repeat-containing protein At1g02060, chloroplastic [*Eucalyptus grandis*]
Pg-miR5077	*Gglean018583.1*	PREDICTED: uncharacterized protein LOC104454281 isoform X3 [*Eucalyptus grandis*]
Pg-miR5077	*Gglean013576.1*	PREDICTED: golgin subfamily A member 4 [*Eucalyptus grandis*]
Pg-miR5077	*Gglean007512.1*	PREDICTED: cytochrome P450 77A2 [*Eucalyptus grandis*]
Pg-miR5152-3p	*Gglean006196.1*	aspartyl protease family protein [*Populus trichocarpa*]
Pg-miR5223	*Gglean001412.1*	hypothetical protein JCGZ_07785 [*Jatropha curcas*]
Pg-miR5570	*Gglean009768.1*	Ypt/Rab-GAP domain of gyp1p superfamily protein isoform 1 [*Theobroma cacao*]
Pg-miR5570	*Gglean004612.1*	PREDICTED: ubiquitin carboxyl-terminal hydrolase 16-like [*Eucalyptus grandis*]
Pg-miR5671a	*Gglean019771.1*	PREDICTED: uncharacterized protein LOC104425114 [*Eucalyptus grandis*]
Pg-miR5671a	*Gglean027773.1*	Uncharacterized protein TCM_015048 [*Theobroma cacao*]
Pg-miR5671a	*Gglean008480.1*	PREDICTED: microtubule-associated protein futsch-like [*Cicer arietinum*]
Pg-miR5671a	*Gglean012058.1*	PREDICTED: ras GTPase-activating protein-binding protein 2 [*Eucalyptus grandis*]
Pg-miR5671a	*Gglean000352.1*	PREDICTED: putative pentatricopeptide repeat-containing protein At5g59200, chloroplastic-like [Fragaria vesca subsp. vesca]
Pg-miR5813	*Gglean003874.1*	PREDICTED: uncharacterized protein LOC104591284 [*Nelumbo nucifera*]
Pg-miR5813	*Gglean002988.1*	PREDICTED: uncharacterized protein C57A7.06 [*Eucalyptus grandis*]
Pg-miR7532a	*Gglean019131.1*	PREDICTED: COBRA-like protein 7 [*Eucalyptus grandis*]
Pg-miR7532a	*Gglean003874.1*	PREDICTED: uncharacterized protein LOC104591284 [*Nelumbo nucifera*]
Pg-miR7532a	*Gglean030660.1*	NA[a]
Pg-miR7532a	*Gglean017505.1*	PREDICTED: probable carboxylesterase 2 [*Eucalyptus grandis*]
Pg-miR7532a	*Gglean023552.1*	PREDICTED: DNA-directed RNA polymerases II, IV and V subunit 8B-like isoform X1 [*Eucalyptus grandis*]
Pg-miR7782-3p	*Gglean027463.1*	PREDICTED: defensin-like protein 182 [*Malus domestica*]
Pg-miR8148	*Gglean025175.1*	NA[a]
Pg-miR827-5p	*Gglean013516.1*	Duplicated homeodomain-like superfamily protein, putative isoform 1 [*Theobroma cacao*]
Pg-miR827-5p	*Gglean022904.1*	PREDICTED: transcription initiation factor TFIID subunit 1 isoform X1 [*Eucalyptus grandis*]

Pg-miR827-5p	*Gglean006537.1*	PREDICTED: MADS-box protein FBP24-like [*Eucalyptus grandis*]
Pg-miR858b	*Gglean019167.1*	PREDICTED: probable 3-ketoacyl-CoA synthase 20 [*Eucalyptus grandis*]
Pg-miR858b	*Gglean004274.1*	PREDICTED: transcription factor WER-like [*Eucalyptus grandis*]
Pg-miR858b	*Gglean020871.1*	PREDICTED: anthocyanin regulatory C1 protein-like [*Eucalyptus grandis*]
Pg-miR858b	*Gglean031077.1*	PREDICTED: zinc finger protein ZAT9-like [*Nicotiana tomentosiformis*]
Pg-miR858b	*Gglean031080.1*	PREDICTED: uncharacterized protein LOC103330234 [*Prunus mume*]
Pg-miR858b	*Gglean004276.1*	PREDICTED: transcription factor WER-like [*Eucalyptus grandis*]
Pg-miR858b	*Gglean027607.1*	PREDICTED: transcription factor MYB12-like [*Glycine max*]
Pg-miR858b	*Gglean008242.1*	myb-related transcription factor [*Panax ginseng*]
Pg-miR858b	*Gglean014090.1*	r2r3-myb transcription factor, putative [*Ricinus communis*]
Pg-miR858b	*Gglean018250.1*	PREDICTED: aldehyde dehydrogenase family 7 member B4 [*Eucalyptus grandis*]
Pg-miR858b	*Gglean023939.1*	PREDICTED: myb-related protein Zm38-like [Prunus mume]
Pg-miR858b	*Gglean029266.1*	hypothetical protein CICLE_v10014958mg [Citrus clementina]
Pg-miR858b	*Gglean004315.1*	Duplicated homeodomain-like superfamily protein [Theobroma cacao]
Pg-miR858b	*Gglean020873.1*	PREDICTED: transcription factor TT2-like [*Eucalyptus grandis*]
Pg-miR858b	*Gglean026143.1*	PREDICTED: myb-related protein Myb4 [Prunus mume]
Pg-miR858b	*Gglean012452.1*	PREDICTED: transcription repressor MYB4 [*Eucalyptus grandis*]
Pg-miR8712	*Gglean026247.1*	unnamed protein product [Nicotiana tabacum]
Pg-miR8712	*Gglean015249.1*	unnamed protein product [Nicotiana tabacum]
Pg-miR8712	*Gglean004616.1*	unnamed protein product [Nicotiana tabacum]
Pg-miR8712	*Gglean024246.1*	retrotransposon-like protein [Musa acuminata]
Pg-miR8712	*Gglean026959.1*	unnamed protein product [Nicotiana tabacum]
Pg-miR8712	*Gglean008852.1*	unnamed protein product [Nicotiana tabacum]
Pg-miR8712	*Gglean019316.1*	hypothetical protein VITISV_007384 [Vitis vinifera]
Pg-miR8712	*Gglean025906.1*	EXS family protein [Theobroma cacao]
Pg-miR8712	*Gglean009062.1*	PREDICTED: sugar transport protein 14 [*Eucalyptus grandis*]
Pg-miR8712	*Gglean015308.1*	unnamed protein product [Nicotiana tabacum]

Pg-miR8712	*Gglean006955.1*	hypothetical retrotransposon [Ipomoea batatas]
Pg-miR902j-5p	*Gglean020069.1*	PREDICTED: ankyrin repeat and SAM domain-containing protein 6-like [*Eucalyptus grandis*]
Pg-miR902j-5p	*Gglean005372.1*	PREDICTED: uncharacterized protein LOC101260488 [*Solanum lycopersicum*]
Pg-miR902j-5p	*Gglean017067.1*	hypothetical protein PRUPE_ppa018570mg, partial [Prunus persica]
Pg-miR902j-5p	*Gglean009864.1*	PREDICTED: uncharacterized protein LOC104418924 [*Eucalyptus grandis*]
Pg-miR952b	*Gglean017511.1*	PREDICTED: presequence protease 2, chloroplastic/mitochondrial-like [*Eucalyptus grandis*]
Pg-miR952b	*Gglean014267.1*	PREDICTED: putative RING-H2 finger protein ATL21A [*Eucalyptus grandis*]
Pg-miR952b	*Gglean014820.1*	PREDICTED: jacalin-related lectin 4-like isoform X2 [*Eucalyptus grandis*]
Pg-miR952b	*Gglean010890.1*	PREDICTED: protein PLANT CADMIUM RESISTANCE 10 [*Eucalyptus grandis*]
Pg-miR952b	*Gglean001502.1*	PREDICTED: uncharacterized protein LOC104445657 [*Eucalyptus grandis*]
Pg-miR952b	*Gglean018134.1*	PREDICTED: uncharacterized protein LOC104454517 isoform X2 [*Eucalyptus grandis*]
Pg-miR952b	*Gglean002174.1*	PREDICTED: aspartate aminotransferase, cytoplasmic-like isoform X1 [*Eucalyptus grandis*]
Pg-miR952b	*Gglean014819.1*	PREDICTED: jacalin-related lectin 4-like isoform X2 [*Eucalyptus grandis*]
Pg-miR952b	*Gglean026783.1*	hypothetical protein CICLE_v10031321mg [Citrus clementina]
Pg-miR9726	*Gglean018517.1*	Putative gag-pol polyprotein, identical [Solanum demissum]
Pg-miR9726	*Gglean007218.1*	F15O4.13 [Arabidopsis thaliana]
Pg-miR9726	*Gglean013231.1*	NA[a]
Pg-miR9726	*Gglean030797.1*	hypothetical protein AMTR_s02100p00009220, partial [Amborella trichopoda]
Pg-miR9761	*Gglean019745.1*	PREDICTED: protein trichome birefringence-like 34 [*Eucalyptus grandis*]
Pg-miR9761	*Gglean021204.1*	PREDICTED: U4/U6 small nuclear ribonucleoprotein PRP4-like protein [*Eucalyptus grandis*]
Pg-miR9761	*Gglean006332.1*	PREDICTED: DNA mismatch repair protein MSH1, mitochondrial isoform X2 [*Eucalyptus grandis*]
Pg-miR9761	*Gglean000301.1*	Cyclic nucleotide-binding transporter 1 isoform 10 [Theobroma cacao]
Pg-miRN01	*Gglean004274.1*	PREDICTED: transcription factor WER-like [*Eucalyptus grandis*]
Pg-miRN01	*Gglean031077.1*	PREDICTED: zinc finger protein ZAT9-like [Nicotiana tomentosiformis]
Pg-miRN01	*Gglean020873.1*	PREDICTED: transcription factor TT2-like [*Eucalyptus grandis*]
Pg-miRN01	*Gglean020871.1*	PREDICTED: anthocyanin regulatory C1 protein-like [*Eucalyptus grandis*]
Pg-miRN01	*Gglean023939.1*	PREDICTED: myb-related protein Zm38-like [Prunus mume]

Pg-miRN01	*Gglean004276.1*	PREDICTED: transcription factor WER-like [*Eucalyptus grandis*]
Pg-miRN01	*Gglean027607.1*	PREDICTED: transcription factor MYB12-like [Glycine max]
Pg-miRN01	*Gglean020505.1*	hypothetical protein EUGRSUZ_D02650 [*Eucalyptus grandis*]
Pg-miRN01	*Gglean008242.1*	myb-related transcription factor [Panax ginseng]
Pg-miRN01	*Gglean012452.1*	PREDICTED: transcription repressor MYB4 [*Eucalyptus grandis*]
Pg-miRN01	*Gglean014090.1*	r2r3-myb transcription factor, putative [Ricinus communis]
Pg-miRN01	*Gglean029266.1*	hypothetical protein CICLE_v10014958mg [Citrus clementina]
Pg-miRN01	*Gglean026143.1*	PREDICTED: myb-related protein Myb4 [Prunus mume]
Pg-miRN01	*Gglean004315.1*	Duplicated homeodomain-like superfamily protein [Theobroma cacao]
Pg-miRN02	*Gglean003203.1*	PREDICTED: probably inactive leucine-rich repeat receptor-like protein kinase At3g28040 [*Eucalyptus grandis*]
Pg-miRN03	*Gglean012701.1*	PREDICTED: uncharacterized protein LOC104417748 [*Eucalyptus grandis*]
Pg-miRN03	*Gglean026508.1*	hypothetical protein JCGZ_06261 [Jatropha curcas]
Pg-miRN03	*Gglean029554.1*	PREDICTED: putative B3 domain-containing protein At3g24850 [Vitis vinifera]
Pg-miRN03	*Gglean017067.1*	hypothetical protein PRUPE_ppa018570mg, partial [Prunus persica]
Pg-miRN03	*Gglean002260.1*	PREDICTED: small glutamine-rich tetratricopeptide repeat-containing protein 2 [Tarenaya hassleriana]
Pg-miRN03	*Gglean011692.1*	hypothetical protein EUGRSUZ_D00484 [*Eucalyptus grandis*]
Pg-miRN06	*Gglean020082.1*	PREDICTED: uncharacterized protein LOC104417739 [*Eucalyptus grandis*]
Pg-miRN07	*Gglean013966.1*	PREDICTED: homeobox-leucine zipper protein ATHB-8-like [*Eucalyptus grandis*]
Pg-miRN07	*Gglean027183.1*	HB1-like protein [Eucalyptus cladocalyx]
Pg-miRN07	*Gglean010679.1*	PREDICTED: 60S ribosomal protein L9 [*Eucalyptus grandis*]
Pg-miRN07	*Gglean031286.1*	DNA binding protein, putative [Ricinus communis]
Pg-miRN10	*Gglean012177.1*	hypothetical protein JCGZ_09223 [Jatropha curcas]
Pg-miRN10	*Gglean031286.1*	DNA binding protein, putative [Ricinus communis]
Pg-miRN10	*Gglean027183.1*	HB1-like protein [Eucalyptus cladocalyx]
Pg-miRN10	*Gglean013966.1*	PREDICTED: homeobox-leucine zipper protein ATHB-8-like [*Eucalyptus grandis*]
Pg-miRN11	*Gglean007369.1*	PREDICTED: nodulation-signaling pathway 2 protein-like [Citrus sinensis]
Pg-miRN11	*Gglean006729.1*	PREDICTED: scarecrow-like protein 6 [*Eucalyptus grandis*]

Pg-miRN11	*Gglean026987.1*	PREDICTED: acid beta-fructofuranosidase-like [*Eucalyptus grandis*]
Pg-miRN11	*Gglean003203.1*	PREDICTED: probably inactive leucine-rich repeat receptor-like protein kinase At3g28040 [*Eucalyptus grandis*]
Pg-miRN11	*Gglean024398.1*	hypothetical protein CICLE_v10006513mg [Citrus clementina]
Pg-miRN11	*Gglean025172.1*	PREDICTED: scarecrow-like protein 15 [*Eucalyptus grandis*]
Pg-miRN11	*Gglean006444.1*	Phosphoenolpyruvate carboxylase, putative [Ricinus communis]
Pg-miRN11	*Gglean025172.1*	PREDICTED: scarecrow-like protein 15 [*Eucalyptus grandis*]
Pg-miRN11	*Gglean027349.1*	PREDICTED: uncharacterized protein LOC105170581 [Sesamum indicum]
Pg-miRN11	*Gglean014673.1*	PREDICTED: SNF1-related protein kinase regulatory subunit beta-1 [Vitis vinifera]
Pg-miRN11	*Gglean004494.1*	PREDICTED: mitochondrial ribosome-associated GTPase 1 isoform X1 [*Eucalyptus grandis*]
Pg-miRN11	*Gglean006729.1*	PREDICTED: scarecrow-like protein 6 [*Eucalyptus grandis*]
Pg-miRN12	*Gglean012868.1*	PREDICTED: WD repeat-containing protein LWD1-like [Cicer arietinum]
Pg-miRN12	*Gglean010888.1*	PREDICTED: F-box protein SKIP24 [*Eucalyptus grandis*]
Pg-miRN12	*Gglean002551.1*	PREDICTED: pentatricopeptide repeat-containing protein At5g46580, chloroplastic [*Eucalyptus grandis*]
Pg-miRN12	*Gglean018747.1*	PREDICTED: uncharacterized protein LOC103332870 [Prunus mume]
Pg-miRN13	*Gglean001744.1*	NA[a]
Pg-miRN14	*Gglean007120.1*	PREDICTED: NAC domain-containing protein 68-like [*Eucalyptus grandis*]
Pg-miRN14	*Gglean007093.1*	PREDICTED: NAC domain-containing protein 89-like [*Eucalyptus grandis*]
Pg-miRN14	*Gglean007118.1*	PREDICTED: NAC domain-containing protein 74-like [*Eucalyptus grandis*]
Pg-miRN14	*Gglean007121.1*	PREDICTED: NAC domain-containing protein 74-like [*Eucalyptus grandis*]
Pg-miRN14	*Gglean020373.1*	NA[a]
Pg-miRN14	*Gglean003378.1*	Os09g0539500 [Oryza sativa Japonica Group]
Pg-miRN15	*Gglean022261.1*	hypothetical protein EUGRSUZ_F02430 [*Eucalyptus grandis*]
Pg-miRN15	*Gglean025452.1*	PREDICTED: uncharacterized protein LOC101315171 [Fragaria vesca subsp. vesca]
Pg-miRN15	*Gglean003665.1*	PREDICTED: serine/threonine-protein kinase BRI1-like 1 [Populus euphratica]
Pg-miRN15	*Gglean000925.1*	PREDICTED: dnaJ homolog subfamily B member 1 [*Eucalyptus grandis*]
Pg-miRN15	*Gglean012251.1*	Leucine-rich repeat receptor protein kinase EXS precursor, putative [Ricinus communis]
Pg-miRN16	*Gglean007121.1*	PREDICTED: NAC domain-containing protein 74-like [*Eucalyptus grandis*]

Pg-miRN16	*Gglean007120.1*	PREDICTED: NAC domain-containing protein 68-like [*Eucalyptus grandis*]
Pg-miRN16	*Gglean007104.1*	PREDICTED: NAC domain-containing protein 89-like [*Eucalyptus grandis*]
Pg-miRN16	*Gglean007118.1*	PREDICTED: NAC domain-containing protein 74-like [*Eucalyptus grandis*]
Pg-miRN16	*Gglean007093.1*	PREDICTED: NAC domain-containing protein 89-like [*Eucalyptus grandis*]
Pg-miRN16	*Gglean020373.1*	NA[a]
Pg-miRN18	*Gglean017814.1*	PREDICTED: transcription factor GAMYB [*Eucalyptus grandis*]
Pg-miRN18	*Gglean013014.1*	Uncharacterized protein F383_17343 [Gossypium arboreum]
Pg-miRN18	*Gglean015155.1*	PREDICTED: protein SENSITIVE TO PROTON RHIZOTOXICITY 1 [*Eucalyptus grandis*]
Pg-miRN18	*Gglean013127.1*	hypothetical protein PRUPE_ppa004612mg [Prunus persica]
Pg-miRN18	*Gglean006071.1*	PREDICTED: uncharacterized protein LOC104450247 [*Eucalyptus grandis*]
Pg-miRN18	*Gglean016054.1*	PREDICTED: transcription factor GAMYB [*Eucalyptus grandis*]
Pg-miRN19	*Gglean000395.1*	PREDICTED: cytochrome b5 [*Eucalyptus grandis*]
Pg-miRN19	*Gglean009790.1*	NA[a]
Pg-miRN19	*Gglean011726.1*	hypothetical protein VOLCADRAFT_98215 [Volvox carteri f. nagariensis]
Pg-miRN19	*Gglean011729.1*	hypothetical protein VOLCADRAFT_98215 [Volvox carteri f. nagariensis]
Pg-miRN19	*Gglean008635.1*	hypothetical protein EUGRSUZ_E03531 [*Eucalyptus grandis*]
Pg-miRN20	*Gglean006537.1*	PREDICTED: MADS-box protein FBP24-like [*Eucalyptus grandis*]
Pg-miRN20	*Gglean022904.1*	PREDICTED: transcription initiation factor TFIID subunit 1 isoform X1 [*Eucalyptus grandis*]
Pg-miRN20	*Gglean013516.1*	Duplicated homeodomain-like superfamily protein, putative isoform 1 [Theobroma cacao]
Pg-miRN21	*Gglean019717.1*	laccase family protein [Populus trichocarpa]
Pg-miRN21	*Gglean026841.1*	PREDICTED: probable inorganic phosphate transporter 1-3 [*Eucalyptus grandis*]
Pg-miRN23	*Gglean014469.1*	PREDICTED: protein ETHYLENE INSENSITIVE 3-like [*Eucalyptus grandis*]
Pg-miRN23	*Gglean000349.1*	NA[a]
Pg-miRN24	*Gglean027183.1*	HB1-like protein [Eucalyptus cladocalyx]
Pg-miRN27	*Gglean026841.1*	PREDICTED: probable inorganic phosphate transporter 1-3 [*Eucalyptus grandis*]
Pg-miRN27	*Gglean024852.1*	PREDICTED: uncharacterized protein LOC104457236 isoform X1 [*Eucalyptus grandis*]
Pg-miRN27	*Gglean019717.1*	laccase family protein [*Populus trichocarpa*]

Pg-miRN28	*Gglean022778.1*	PREDICTED: ATP sulfurylase 1, chloroplastic-like [*Eucalyptus grandis*]
Pg-miRN28	*Gglean015303.1*	Early nodulin 70 family protein [*Populus trichocarpa*]
Pg-miRN28	*Gglean006692.1*	NA[a]
Pg-miRN29	*Gglean014871.1*	Domain of Uncharacterized protein function isoform 1 [*Theobroma cacao*]
Pg-miRN29	*Gglean000420.1*	PREDICTED: glucose-6-phosphate 1-dehydrogenase, chloroplastic-like [*Eucalyptus grandis*]
Pg-miRN29	*Gglean013301.1*	NB-ARC domain-containing disease resistance protein, putative [*Theobroma cacao*]
Pg-miRN30	*Gglean020813.1*	NA[a]
Pg-miRN30	*Gglean024339.1*	NA[a]
Pg-miRN30	*Gglean016215.1*	60S ribosomal protein L17-2 [*Morus notabilis*]
Pg-miRN30	*Gglean017261.1*	NA[a]
Pg-miRN30	*Gglean006990.1*	NA[a]
Pg-miRN30	*Gglean006409.1*	NA[a]
Pg-miRN30	*Gglean015850.1*	Protein kinase-like domain protein [Ophiocordyceps sinensis CO18]
Pg-miRN30	*Gglean017324.1*	ICE transcription factor 1 [*Eucalyptus globulus*]
Pg-miRN30	*Gglean018371.1*	NA[a]
Pg-miRN30	*Gglean011392.1*	PREDICTED: pentatricopeptide repeat-containing protein At4g25270, chloroplastic [*Eucalyptus grandis*]
Pg-miRN30	*Gglean000351.1*	PREDICTED: putative pentatricopeptide repeat-containing protein At5g59200, chloroplastic [*Prunus mume*]
Pg-miRN30	*Gglean030793.1*	NA[a]
Pg-miRN30	*Gglean023371.1*	NA[a]
Pg-miRN30	*Gglean013578.1*	hypothetical protein EUGRSUZ_B01521 [*Eucalyptus grandis*]
Pg-miRN30	*Gglean012173.1*	NA[a]
Pg-miRN30	*Gglean026569.1*	NA[a]
Pg-miRN30	*Gglean015977.1*	NA[a]
Pg-miRN30	*Gglean027274.1*	NA[a]
Pg-miRN30	*Gglean003916.1*	hypothetical protein L484_027736 [*Morus notabilis*]
Pg-miRN30	*Gglean027182.1*	NA[a]
Pg-miRN31	*Gglean004741.1*	unnamed protein product [*Vitis vinifera*]

Pg-miRN31	*Gglean028306.1*	LIGULELESS1 protein, putative [*Theobroma cacao*]
Pg-miRN31	*Gglean012943.1*	unnamed protein product [*Vitis vinifera*]
Pg-miRN31	*Gglean028488.1*	PREDICTED: squamosa promoter-binding-like protein 13A [*Vitis vinifera*]
Pg-miRN31	*Gglean016208.1*	PREDICTED: squamosa promoter-binding-like protein 2 [*Eucalyptus grandis*]
Pg-miRN31	*Gglean000847.1*	LIGULELESS1 protein, putative [*Theobroma cacao*]
Pg-miRN31	*Gglean016197.1*	PREDICTED: squamosa promoter-binding-like protein 16 [*Vitis vinifera*]
Pg-miRN33	*Gglean016197.1*	PREDICTED: squamosa promoter-binding-like protein 16 [*Vitis vinifera*]
Pg-miRN33	*Gglean000847.1*	LIGULELESS1 protein, putative [*Theobroma cacao*]
Pg-miRN33	*Gglean001912.1*	PREDICTED: squamosa promoter-binding-like protein 7 [*Eucalyptus grandis*]
Pg-miRN33	*Gglean004741.1*	unnamed protein product [*Vitis vinifera*]
Pg-miRN33	*Gglean028306.1*	LIGULELESS1 protein, putative [*Theobroma cacao*]
Pg-miRN33	*Gglean016208.1*	PREDICTED: squamosa promoter-binding-like protein 2 [*Eucalyptus grandis*]
Pg-miRN33	*Gglean028488.1*	PREDICTED: squamosa promoter-binding-like protein 13A [*Vitis vinifera*]
Pg-miRN35	*Gglean020373.1*	NA[a]
Pg-miRN35	*Gglean010802.1*	RING/U-box superfamily protein isoform 1 [*Theobroma cacao*]
Pg-miRN35	*Gglean007104.1*	PREDICTED: NAC domain-containing protein 89-like [*Eucalyptus grandis*]
Pg-miRN36	*Gglean003827.1*	PREDICTED: WD repeat-containing protein 44-like [*Malus domestica*]
Pg-miRN36	*Gglean007369.1*	PREDICTED: nodulation-signaling pathway 2 protein-like [*Citrus sinensis*]
Pg-miRN36	*Gglean029630.1*	PREDICTED: BRCT domain-containing protein At4g02110 [*Eucalyptus grandis*]
Pg-miRN38	*Gglean017109.1*	auxin signaling f-box 2 -like protein [*Gossypium arboreum*]
Pg-miRN38	*Gglean006519.1*	PREDICTED: protein TRANSPORT INHIBITOR RESPONSE 1 [*Vitis vinifera*]
Pg-miRN40	*Gglean029526.1*	PREDICTED: phenazine biosynthesis-like domain-containing protein 1 [*Eucalyptus grandis*]
Pg-miRN40	*Gglean004741.1*	unnamed protein product [*Vitis vinifera*]
Pg-miRN40	*Gglean005913.1*	PREDICTED: ATPase WRNIP1 [*Eucalyptus grandis*]
Pg-miRN40	*Gglean016208.1*	PREDICTED: squamosa promoter-binding-like protein 2 [*Eucalyptus grandis*]
Pg-miRN40	*Gglean028488.1*	PREDICTED: squamosa promoter-binding-like protein 13A [*Vitis vinifera*]
Pg-miRN40	*Gglean028306.1*	LIGULELESS1 protein, putative [*Theobroma cacao*]

Pg-miRN40	*Gglean016197.1*	PREDICTED: squamosa promoter-binding-like protein 16 [*Vitis vinifera*]
Pg-miRN40	*Gglean000847.1*	LIGULELESS1 protein, putative [*Theobroma cacao*]
Pg-miRN40	*Gglean001912.1*	PREDICTED: squamosa promoter-binding-like protein 7 [*Eucalyptus grandis*]
Pg-miRN45	*Gglean026885.1*	LAC [*Punica granatum*]
Pg-miRN45	*Gglean021591.1*	Laccase/Diphenol oxidase family protein isoform 1 [*Theobroma cacao*]
Pg-miRN45	*Gglean004768.1*	PREDICTED: laccase-17-like [*Eucalyptus grandis*]
Pg-miRN45	*Gglean001274.1*	PREDICTED: monogalactosyldiacylglycerol synthase, chloroplastic isoform X1 [*Eucalyptus grandis*]
Pg-miRN45	*Gglean021685.1*	PREDICTED: laccase-7 [*Vitis vinifera*]
Pg-miRN45	*Gglean021664.1*	PREDICTED: laccase-17-like [*Eucalyptus grandis*]
Pg-miRN45	*Gglean019708.1*	PREDICTED: laccase-7 [*Eucalyptus grandis*]
Pg-miRN45	*Gglean030878.1*	hypothetical protein JCGZ_08506 [*Jatropha curcas*]
Pg-miRN45	*Gglean021592.1*	PREDICTED: laccase-4-like [*Eucalyptus grandis*]
Pg-miRN45	*Gglean023503.1*	PREDICTED: laccase-17-like [Cicer arietinum]
Pg-miRN45	*Gglean019717.1*	laccase family protein [*Populus trichocarpa*]
Pg-miRN45	*Gglean011766.1*	PREDICTED: 21 kDa protein-like [*Eucalyptus grandis*]
Pg-miRN45	*Gglean006292.1*	PREDICTED: laccase-17-like [*Cicer arietinum*]
Pg-miRN45	*Gglean014191.1*	PREDICTED: laccase-17 [*Sesamum indicum*]
Pg-miRN45	*Gglean029998.1*	hypothetical protein CICLE_v10011358mg [*Citrus clementina*]
Pg-miRN45	*Gglean019707.1*	PREDICTED: laccase-7-like [*Eucalyptus grandis*]
Pg-miRN46	*Gglean012201.1*	PREDICTED: uncharacterized protein LOC102667057 [*Glycine max*]
Pg-miRN46	*Gglean005513.1*	NA[a]
Pg-miRN46	*Gglean013950.1*	PREDICTED: leucine-rich repeat extensin-like protein 3-like [*Glycine max*]
Pg-miRN46	*Gglean020212.1*	PREDICTED: protein NSP-INTERACTING KINASE 3 [*Eucalyptus grandis*]
Pg-miRN46	*Gglean017542.1*	PREDICTED: pollen-specific leucine-rich repeat extensin-like protein 3 [*Eucalyptus grandis*]
Pg-miRN46	*Gglean022560.1*	PREDICTED: probable metal-nicotianamine transporter YSL5-like [*Citrus sinensis*]
Pg-miRN47	*Gglean016608.1*	NA[a]
Pg-miRN48	*Gglean011726.1*	hypothetical protein VOLCADRAFT_98215 [*Volvox carteri f. nagariensis*]

Pg-miRN48	*Gglean011729.1*	hypothetical protein VOLCADRAFT_98215 [*Volvox carteri f. nagariensis*]
Pg-miRN49	*Gglean008604.1*	PREDICTED: cytochrome P450 734A1 [*Eucalyptus grandis*]
Pg-miRN51	*Gglean024398.1*	hypothetical protein CICLE_v10006513mg [*Citrus clementina*]
Pg-miRN53	*Gglean020297.1*	PREDICTED: F-box only protein 6 [*Eucalyptus grandis*]
Pg-miRN53	*Gglean014016.1*	PREDICTED: serine/threonine-protein kinase BLUS1-like isoform X1 [*Eucalyptus grandis*]
Pg-miRN56	*Gglean022642.1*	hypothetical protein L484_021218 [*Morus notabilis*]
Pg-miRN56	*Gglean007834.1*	hypothetical protein PRUPE_ppa019065mg, partial [*Prunus persica*]
Pg-miRN56	*Gglean003381.1*	hypothetical protein PRUPE_ppa008801mg [*Prunus persica*]
Pg-miRN56	*Gglean003444.1*	PREDICTED: protein CUP-SHAPED COTYLEDON 2 [*Eucalyptus grandis*]
Pg-miRN56	*Gglean026964.1*	PREDICTED: UDP-glucuronic acid decarboxylase 6-like [*Populus euphratica*]

Note: a: genes without annotation

TableS7 Gene ontology categories of differentially expressed miRNAs' target genes

GO_accession	term_type	Term	Query item	Query total	refitem	reftotal	pvalue	FDR
GO:0046274	Biological Process	lignin catabolic process	11	69	33	13986	1.80E-17	3.70E-15
GO:0046271	Biological Process	phenylpropanoid catabolic process	11	69	33	13986	1.80E-17	3.70E-15
GO:0019439	Biological Process	aromatic compound catabolic process	11	69	59	13986	2.30E-14	3.10E-12
GO:0042219	Biological Process	cellular amino acid derivative catabolic process	11	69	66	13986	8.30E-14	6.90E-12
GO:0009808	Biological Process	lignin metabolic process	11	69	66	13986	8.30E-14	6.90E-12
GO:0009698	Biological Process	phenylpropanoid metabolic process	11	69	165	13986	2.00E-09	1.40E-07
GO:0048608	Biological Process	reproductive structure development	15	69	578	13986	9.30E-07	5.00E-05
GO:0006725	Biological Process	cellular aromatic compound metabolic process	13	69	435	13986	9.60E-07	5.00E-05
GO:0006575	Biological Process	cellular amino acid derivative metabolic process	11	69	309	13986	1.10E-06	5.20E-05
GO:0003006	Biological Process	reproductive developmental process	15	69	659	13986	4.40E-06	0.00018
GO:0044282	Biological Process	small molecule catabolic process	12	69	471	13986	1.10E-05	0.00043

GO:0009791	Biological Process	post-embryonic development	15	69	729	13986	1.40E-05	0.00048
GO:0022414	Biological Process	reproductive process	15	69	740	13986	1.70E-05	0.00053
GO:0000003	Biological Process	reproduction	15	69	759	13986	2.20E-05	0.00065
GO:0019748	Biological Process	secondary metabolic process	11	69	490	13986	7.60E-05	0.0021
GO:0048438	Biological Process	floral whorl development	6	69	140	13986	9.80E-05	0.0025
GO:0048437	Biological Process	floral organ development	6	69	168	13986	0.00026	0.0064
GO:0048569	Biological Process	post-embryonic organ development	6	69	170	13986	0.00028	0.0064
GO:0010016	Biological Process	shoot morphogenesis	6	69	182	13986	0.0004	0.0087
GO:0005507	Mollecular Function	copper ion binding	12	69	159	13986	9.70E-11	7.40E-09
GO:0008447	Mollecular Function	L-ascorbate oxidase activity	5	69	23	13986	1.10E-07	4.30E-06
GO:0016682	Mollecular Function	oxidoreductase activity, acting on diphenols and related substances as donors, oxygen as acceptor	5	69	40	13986	2.00E-06	5.20E-05
GO:0003700	Mollecular Function	transcription factor activity	8	69	168	13986	3.50E-06	5.60E-05

GO:0016679	Mollecular Function	oxidoreductase activity, acting on diphenols and related substances as donors	5	69	45	13986	3.70E-06	5.60E-05
GO:0030528	Mollecular Function	transcription regulator activity	8	69	194	13986	1.00E-05	0.00013
GO:0043167	Mollecular Function	ion binding	25	69	2106	13986	0.00033	0.0037
GO:0046914	Mollecular Function	transition metal ion binding	16	69	1127	13986	0.00048	0.0046
GO:0003677	Mollecular Function	DNA binding	11	69	642	13986	0.00071	0.006
GO:0046872	Mollecular Function	metal ion binding	23	69	2009	13986	0.00081	0.0062
GO:0043169	Mollecular Function	cation binding	23	69	2050	13986	0.001	0.0072
GO:0048046	Cellular Component	apoplast	12	69	190	13986	7.50E-10	3.70E-08
GO:0005576	Cellular Component	extracellular region	12	69	382	13986	1.40E-06	3.50E-05

Table S8 Differentially expressed miRNAs and their corresponding targets extracted from RNA-seq

miRNA	log2FC(ATNSI/TNSI)	log2FC(ATNSII/TNSII)	log2FC(ATNSIII/TNSIII)	log2FC(ATNSII/ATNSI)	log2FC(ATNSIII/ATNSI)	log2FC(ATNSIII/ATNSII)	log2FC(TNSII/TNSI)	log2FC(TNSIII/TNSI)	log2FC(TNSIII/TNSII)	Target	log2FC(ATNSI/TNSI)	log2FC(ATNSII/TNSII)	log2FC(ATNSIII/TNSIII)	log2FC (ATNSII/ATNSI)	log2FC (ATNSIII/ATNSI)	log2FC (ATNSIII/ATNSII)	log2FC (TNSII/TNSI)	log2FC (TNSIII/TNSI)	log2FC (TNSIII/TNSII)	Annotation
miR156c	1.94	-0.17	-2.32	1.90	0.88	-0.99	3.98	5.00	0.99	*Gglean028306.1*	-0.02	-0.05	-0.17	0.25	-1.35	-1.60	0.30	-1.21	-1.48	LIGULELESS1 protein, putative [*Theobroma cacao*]
miR156c	1.94	-0.17	-2.32	1.90	0.88	-0.99	3.98	5.00	0.99	*Gglean016197.1*	-0.20	-0.08	-0.84	0.06	-2.10	-2.15	-0.04	-1.46	-1.40	PREDICTED: squamosa promoter-binding-like protein 16 [*Vitis vinifera*]
miR156c	1.94	-0.17	-2.32	1.90	0.88	-0.99	3.98	5.00	0.99	*Gglean028488.1*	0.16	-0.78	-0.80	-1.69	-2.69	-0.99	-0.74	-1.75	-0.98	PREDICTED: squamosa promoter-binding-like protein 13A [*Vitis vinifera*]
miR156c	1.94	-0.17	-2.32	1.90	0.88	-0.99	3.98	5.00	0.99	*Gglean000847.1*	-0.14	0.32	-0.05	0.14	-0.36	-0.49	-0.31	-0.46	-0.12	PREDICTED: *Vitis vinifera* squamosa promoter-binding-like protein 6 (LOC100245772), transcript variant X2, mRNA
miR156c	1.94	-0.17	-2.32	1.90	0.88	-0.99	3.98	5.00	0.99	*Gglean001912.1*	0.32	-1.23	0.00	-2.47	-6.81	-4.33	-0.90	-4.73	-3.80	PREDICTED: Eucalyptus grandis squamosa promoter-binding-like protein 7 (LOC104442282), mRNA
miR156c	1.94	-0.17	-2.32	1.90	0.88	-0.99	3.98	5.00	0.99	*Gglean016208.1*	-0.07	0.49	0.27	0.00	0.26	-0.42	0.14	-0.09	-0.20	PREDICTED: squamosa promoter-binding-like protein 2 [*Eucalyptus grandis*]
miR156c	1.94	-0.17	-2.32	1.90	0.88	-0.99	3.98	5.00	0.99	*Gglean004741.1*	-0.11	-0.01	-0.20	-0.16	-2.65	-2.48	-0.25	-2.57	-2.31	PREDICTED: *Eucalyptus grandis* squamosa promoter-binding-like protein 9 (LOC104445761), mRNA
miR157a-5p	-0.92	-0.72	-1.65	-0.10	-0.18	-0.03	-0.26	0.49	0.72	*Gglean016208.1*	-0.07	0.49	0.27	0.00	0.26	-0.42	0.14	-0.09	-0.20	PREDICTED: squamosa promoter-binding-like protein 2 [*Eucalyptus grandis*]
miR157a-5p	-0.92	-0.72	-1.65	-0.10	-0.18	-0.03	-0.26	0.49	0.72	*Gglean000847.1*	-0.14	0.32	-0.05	0.14	-0.36	-0.49	-0.31	-0.46	-0.12	PREDICTED: *Vitis vinifera* squamosa promoter-binding-like protein 6 (LOC100245772), transcript variant X2, mRNA

miR157 a-5p	-0.92	-0.72	-1.65	-0.10	-0.18	-0.03	-0.26	0.49	0.72	*Gglean016197.1*	-0.20	-0.08	-0.84	0.06	-2.10	-2.15	-0.04	-1.46	-1.40	PREDICTED: squamosa promoter-binding-like protein 16 [*Vitis vinifera*]
miR157 a-5p	-0.92	-0.72	-1.65	-0.10	-0.18	-0.03	-0.26	0.49	0.72	*Gglean004741.1*	-0.11	-0.01	-0.20	-0.16	-2.65	-2.48	-0.25	-2.57	-2.31	PREDICTED: Eucalyptus grandis squamosa promoter-binding-like protein 9 (LOC104445761), mRNA
miR157 a-5p	-0.92	-0.72	-1.65	-0.10	-0.18	-0.03	-0.26	0.49	0.72	*Gglean028488.1*	0.16	-0.78	-0.80	-1.69	-2.69	-0.99	-0.74	-1.75	-0.98	PREDICTED: squamosa promoter-binding-like protein 13A [*Vitis vinifera*]
miR157 a-5p	-0.92	-0.72	-1.65	-0.10	-0.18	-0.03	-0.26	0.49	0.72	*Gglean028306.1*	-0.02	-0.05	-0.17	0.25	-1.35	-1.60	0.30	-1.21	-1.48	PREDICTED: *Eucalyptus grandis* squamosa promoter-binding-like protein 6 (LOC104420963), mRNA
miR157 a-5p	-0.92	-0.72	-1.65	-0.10	-0.18	-0.03	-0.26	0.49	0.72	*Gglean012943.1*	-0.07	0.58	0.26	0.96	0.57	-0.38	0.31	0.22	-0.06	Putative uncharacterized protein {ECO:0000313|EMBL:CBI24123.3} OS=*Vitis vinifera* (Grape) PE=4 SV=1
miR165 a-3p	-0.16	1.86	0.25	0.96	1.07	0.17	-1.06	0.54	1.60	*Gglean031286.1*	-0.14	-0.42	-0.76	-0.32	-1.26	-0.93	-0.03	-0.66	-0.60	DNA binding protein, putative [*Ricinus communis*]
miR165 a-3p	-0.16	1.86	0.25	0.96	1.07	0.17	-1.06	0.54	1.60	*Gglean027183.1*	0.07	-0.77	-0.33	-1.29	-1.73	-0.42	-0.45	-1.34	-0.86	PREDICTED: *Eucalyptus grandis* homeobox-leucine zipper protein ATHB-15 (LOC104449909), mRNA
miR165 a-3p	-0.16	1.86	0.25	0.96	1.07	0.17	-1.06	0.54	1.60	*Gglean013966.1*	0.10	-1.19	-1.22	-2.15	-3.57	-1.41	-0.85	-2.26	-1.39	PREDICTED: *Eucalyptus grandis* homeobox-leucine zipper protein ATHB-8-like (LOC104436260), mRNA
miR165 a-3p	-0.16	1.86	0.25	0.96	1.07	0.17	-1.06	0.54	1.60	*Gglean012177.1*	0.17	-1.10	-1.61	-1.79	-3.04	-1.24	-0.51	-1.26	-0.73	PREDICTED: *Eucalyptus grandis* homeobox-leucine zipper protein HOX32 (LOC104440709), transcript variant X2, mRNA
miR166 a-3p	-0.06	0.45	0.72	0.43	0.83	0.44	-0.09	-0.05	0.01	*Gglean012177.1*	0.17	-1.10	-1.61	-1.79	-3.04	-1.24	-0.51	-1.26	-0.73	PREDICTED: *Eucalyptus grandis* homeobox-leucine zipper protein HOX32 (LOC104440709), transcript variant X2, mRNA

miR166a-3p	-0.06	0.45	0.72	0.43	0.83	0.44	-0.09	-0.05	0.01	*Gglean013966.1*	0.10	-1.19	-1.22	-2.15	-3.57	-1.41	-0.85	-2.26	-1.39	PREDICTED: *Eucalyptus grandis* homeobox-leucine zipper protein ATHB-8-like (LOC104436260), mRNA
miR166a-3p	-0.06	0.45	0.72	0.43	0.83	0.44	-0.09	-0.05	0.01	*Gglean027183.1*	0.07	-0.77	-0.33	-1.29	-1.73	-0.42	-0.45	-1.34	-0.86	PREDICTED: *Eucalyptus grandis* homeobox-leucine zipper protein ATHB-15 (LOC104449909), mRNA
miR166a-3p	-0.06	0.45	0.72	0.43	0.83	0.44	-0.09	-0.05	0.01	*Gglean031286.1*	-0.14	-0.42	-0.76	-0.32	-1.26	-0.93	-0.03	-0.66	-0.60	*Ricinus communis* DNA binding protein, putative, mRNA
miR1077-5p	NA[b]	0.42	12.86	NA[b]	1.55	0.18	1.60	NA[b]	-12.33	*Gglean016825.1*	0.00	0.00	0.00	0.00	0.00	0.00	0.00	0.00	0.00	F15O4.13 [*Arabidopsis thaliana*]
miR1077-5p	NA[b]	0.42	12.86	NA[b]	1.55	0.18	1.60	NA[b]	-12.33	*Gglean021499.1*	0.00	0.00	0.00	0.00	0.00	0.00	0.00	0.00	0.00	unknown protein [*Oryza sativa* Japonica Group]
miR167h	-0.69	-0.34	-1.86	1.00	0.86	-0.14	0.63	1.93	1.28	*Gglean022780.1*	-0.11	-0.54	-0.28	-0.46	0.01	0.49	-0.02	0.17	0.22	PREDICTED: *Eucalyptus grandis* probable phosphoribosylformylglycinamidine synthase, chloroplastic/mitochondrial (LOC104418123), mRNA
miR393h	-1.09	0.06	1.00	2.13	2.98	0.89	0.93	0.75	-0.21	*Gglean017109.1*	0.03	0.28	0.07	0.07	-0.35	-0.41	-0.17	-0.39	-0.20	auxin signaling f-box 2 -like protein [*Gossypium arboreum*]
miR393h	-1.09	0.06	1.00	2.13	2.98	0.89	0.93	0.75	-0.21	*Gglean006519.1*	-0.15	0.26	-0.27	0.36	-0.33	-0.68	-0.03	-0.22	-0.16	PREDICTED: protein TRANSPORT INHIBITOR RESPONSE 1 [*Vitis vinifera*]
miR397a	-0.35	5.62	-0.63	5.17	-0.06	-5.13	-0.72	0.13	0.80	*Gglean026885.1*	0.63	0.25	-3.79	0.37	3.41	3.05	0.76	7.81	7.10	LAC [*Punica granatum*]
miR397a	-0.35	5.62	-0.63	5.17	-0.06	-5.13	-0.72	0.13	0.80	*Gglean030438.1*	0.00	-0.99	-1.19	0.00	0.00	0.00	0.48	0.51	0.07	Laccase 3 [*Theobroma cacao*]
miR397a	-0.35	5.62	-0.63	5.17	-0.06	-5.13	-0.72	0.13	0.80	*Gglean021592.1*	0.00	0.00	-3.30	0.00	6.50	4.35	0.00	9.65	6.72	PREDICTED: laccase-4-like [*Eucalyptus grandis*]

miR397a	-0.35	5.62	-0.63	5.17	-0.06	-5.13	-0.72	0.13	0.80	*Gglean021664.1*	0.00	0.00	-3.21	0.00	0.00	0.00	0.00	4.57	3.82	PREDICTED: laccase-17-like [*Eucalyptus grandis*]
miR397a	-0.35	5.62	-0.63	5.17	-0.06	-5.13	-0.72	0.13	0.80	*Gglean019707.1*	0.00	0.00	0.00	0.00	0.00	0.00	0.00	0.00	0.00	PREDICTED: laccase-7-like [*Eucalyptus grandis*]
miR397a	-0.35	5.62	-0.63	5.17	-0.06	-5.13	-0.72	0.13	0.80	*Gglean021685.1*	0.00	0.00	0.00	0.00	0.00	0.00	0.00	0.00	0.00	PREDICTED: laccase-7 [*Vitis vinifera*]
miR397a	-0.35	5.62	-0.63	5.17	-0.06	-5.13	-0.72	0.13	0.80	*Gglean014191.1*	-0.50	-0.54	-2.03	1.52	3.18	1.67	1.56	4.69	3.17	PREDICTED: laccase-17 [*Sesamum indicum*]
miR397a	-0.35	5.62	-0.63	5.17	-0.06	-5.13	-0.72	0.13	0.80	*Gglean018490.1*	0.19	-0.22	-2.60	2.02	5.41	3.40	2.44	8.19	5.79	LAC [*Punica granatum*]
miR397a	-0.35	5.62	-0.63	5.17	-0.06	-5.13	-0.72	0.13	0.80	*Gglean019717.1*	-0.22	-0.11	-3.05	1.43	3.05	1.64	1.33	5.88	4.59	laccase family protein [*Populus trichocarpa*]
miR397a	-0.35	5.62	-0.63	5.17	-0.06	-5.13	-0.72	0.13	0.80	*Gglean009496.1*	-0.16	0.00	0.14	0.02	0.30	0.29	-0.13	-0.01	0.14	PREDICTED: bark storage protein A-like [*Vitis vinifera*]
miR397a	-0.35	5.62	-0.63	5.17	-0.06	-5.13	-0.72	0.13	0.80	*Gglean021591.1*	0.00	0.00	-3.75	0.00	10.22	5.29	3.01	11.77	9.32	Laccase/Diphenol oxidase family protein isoform 1 [*Theobroma cacao*]
miR397a	-0.35	5.62	-0.63	5.17	-0.06	-5.13	-0.72	0.13	0.80	*Gglean019708.1*	-0.54	2.32	1.92	5.84	3.24	-2.59	0.13	0.79	-2.19	PREDICTED: laccase-7 [*Eucalyptus grandis*]
miR398b	-0.15	2.42	4.01	3.60	4.79	1.22	1.03	0.54	-0.52	*Gglean005184.1*	0.00	4.23	3.23	5.29	5.29	0.02	-0.52	0.00	0.00	PREDICTED: purple acid phosphatase 22-like isoform X1 [*Eucalyptus grandis*]
miR4376	-0.70	0.66	1.66	2.48	3.57	1.12	1.12	1.11	-0.03	*Gglean025376.1*	0.00	0.00	0.00	0.00	0.00	0.00	0.00	0.00	0.00	PREDICTED: uncharacterized protein LOC103696520 [*Phoenix dactylifera*]
miR4376	-0.70	0.66	1.66	2.48	3.57	1.12	1.12	1.11	-0.03	*Gglean014835.1*	0.00	-0.17	0.01	-0.06	0.18	0.25	0.00	0.17	0.07	PREDICTED: uncharacterized methyltransferase C3H7.11-like isoform X1 [*Eucalyptus grandis*]

miR444b.1	-6.52	8.69	-8.26	26.41	NA[b]	NA[b]	-1.26	1.58	2.74	*Gglean003233.1*	-0.07	-3.39	-2.44	-3.85	-3.81	0.05	0.00	-1.45	-0.92	PREDICTED: putative leucine-rich repeat-containing protein DDB_G0290503 [*Eucalyptus grandis*]
miR444b.1	-6.52	8.69	-8.26	26.41	NA[b]	NA[b]	-1.26	1.58	2.74	*Gglean004566.1*	0.00	1.24	0.00	2.84	0.00	-2.18	0.00	0.00	0.00	PREDICTED: MADS-box transcription factor 23-like isoform X1 [*Eucalyptus grandis*]
miR5054	-1.40	-0.11	8.95	7.32	8.84	1.59	6.03	-1.62	-7.76	*Gglean029036.1*	-0.47	-0.09	-0.93	0.91	0.17	-0.73	0.54	0.62	0.11	homeobox protein, putative [Ricinus communis]
miR5054	-1.40	-0.11	8.95	7.32	8.84	1.59	6.03	-1.62	-7.76	*Gglean022002.1*	-0.06	0.48	0.84	1.32	2.02	0.71	0.79	1.12	0.35	PREDICTED: scarecrow-like protein 8 [*Eucalyptus grandis*]
miR5054	-1.40	-0.11	8.95	7.32	8.84	1.59	6.03	-1.62	-7.76	*Gglean020952.1*	0.43	1.04	0.93	-0.14	0.38	0.53	-0.74	-0.12	0.65	PREDICTED: probable sarcosine oxidase [*Eucalyptus grandis*]
miR5077	1.51	-0.04	1.28	-0.64	-0.72	-0.02	0.87	-0.62	-1.49	*Gglean007189.1*	0.13	-0.04	0.07	-0.18	-0.11	0.08	0.00	-0.06	-0.03	PREDICTED: pentatricopeptide repeat-containing protein At1g02060, chloroplastic [*Eucalyptus grandis*]
miR5077	1.51	-0.04	1.28	-0.64	-0.72	-0.02	0.87	-0.62	-1.49	*Gglean018583.1*	0.25	0.29	0.17	0.28	-0.08	-0.35	0.25	0.00	-0.22	PREDICTED: uncharacterized protein LOC104454281 isoform X3 [*Eucalyptus grandis*]
miR5077	1.51	-0.04	1.28	-0.64	-0.72	-0.02	0.87	-0.62	-1.49	*Gglean013576.1*	0.00	-0.01	-0.01	0.11	0.20	0.10	0.13	0.21	0.10	PREDICTED: golgin subfamily A member 4 [*Eucalyptus grandis*]
miR5077	1.51	-0.04	1.28	-0.64	-0.72	-0.02	0.87	-0.62	-1.49	*Gglean007512.1*	-0.18	-3.80	-6.92	-4.21	-6.32	-2.10	-0.57	0.40	1.01	PREDICTED: cytochrome P450 77A2 [*Eucalyptus grandis*]
miR5152-3p	-0.07	0.29	9.71	0.49	1.05	0.58	0.05	-8.88	-8.94	*Gglean006196.1*	0.05	0.63	0.40	0.50	0.61	0.12	-0.07	0.25	0.35	aspartyl protease family protein [*Populus trichocarpa*]
miR5223	-0.43	-0.32	-9.10	-0.07	-8.93	-8.82	-0.16	-0.34	-0.22	*Gglean001412.1*	0.08	-0.06	0.46	-0.11	-0.68	-0.56	0.04	-1.07	-1.08	hypothetical protein JCGZ_07785 [*Jatropha curcas*]
miR5671a	-12.30	12.18	0.53	12.03	23.45	-1.26	-12.46	-2.21	23.49	*Gglean019771.1*	-0.40	1.84	1.32	2.95	3.38	0.45	0.71	1.65	0.96	PREDICTED: uncharacterized protein LOC104425114 [*Eucalyptus grandis*]

miR5671a	-12.30	12.18	0.53	12.03	23.45	-1.26	-12.46	-2.21	23.49	*Gglean027773.1*	-0.27	-0.68	-2.82	-1.11	-3.94	-2.83	-0.69	-1.41	-0.69	Uncharacterized protein TCM_015048 [*Theobroma cacao*]
miR5671a	-12.30	12.18	0.53	12.03	23.45	-1.26	-12.46	-2.21	23.49	*Gglean008480.1*	-0.34	3.52	1.68	5.03	5.27	0.25	1.18	3.25	2.11	PREDICTED: microtubule-associated protein futsch-like [*Cicer arietinum*]
miR5671a	-12.30	12.18	0.53	12.03	23.45	-1.26	-12.46	-2.21	23.49	*Gglean012058.1*	-0.04	-0.12	0.23	-0.23	0.13	0.37	-0.13	-0.14	0.02	PREDICTED: ras GTPase-activating protein-binding protein 2 [*Eucalyptus grandis*]
miR858b	0.92	-0.86	0.38	-0.73	-0.31	0.44	1.06	0.14	-0.95	*Gglean004274.1*	-0.49	-0.39	-0.70	0.62	-0.45	-1.06	0.55	-0.23	-0.76	PREDICTED: transcription factor WER-like [*Eucalyptus grandis*]
miR858b	0.92	-0.86	0.38	-0.73	-0.31	0.44	1.06	0.14	-0.95	*Gglean004276.1*	0.22	-0.79	-3.64	-0.83	-1.16	-0.32	0.18	2.69	2.53	PREDICTED: transcription factor WER-like [*Eucalyptus grandis*]
miR858b	0.92	-0.86	0.38	-0.73	-0.31	0.44	1.06	0.14	-0.95	*Gglean027607.1*	-0.52	-0.59	0.13	0.18	1.26	1.09	0.27	0.60	0.36	PREDICTED: transcription factor MYB12-like [*Glycine max*]
miR858b	0.92	-0.86	0.38	-0.73	-0.31	0.44	1.06	0.14	-0.95	*Gglean008242.1*	3.10	2.75	3.18	2.96	1.83	-1.11	0.00	0.00	-1.54	myb-related transcription factor [*Panax ginseng*]
miR858b	0.92	-0.86	0.38	-0.73	-0.31	0.44	1.06	0.14	-0.95	*Gglean018250.1*	0.11	2.37	1.75	3.23	2.56	-0.66	0.98	0.91	-0.03	PREDICTED: aldehyde dehydrogenase family 7 member B4 [*Eucalyptus grandis*]
miR858b	0.92	-0.86	0.38	-0.73	-0.31	0.44	1.06	0.14	-0.95	*Gglean023939.1*	0.00	2.16	0.06	3.34	4.36	1.03	1.08	4.18	3.12	PREDICTED: myb-related protein Zm38-like [*Prunus mume*]
miR858b	0.92	-0.86	0.38	-0.73	-0.31	0.44	1.06	0.14	-0.95	*Gglean004315.1*	-0.15	2.77	1.00	7.29	4.16	-3.12	4.39	3.02	-1.35	Duplicated homeodomain-like superfamily protein [*Theobroma cacao*]
miR858b	0.92	-0.86	0.38	-0.73	-0.31	0.44	1.06	0.14	-0.95	*Gglean012452.1*	-2.68	-1.71	-2.92	3.61	3.60	0.00	2.65	3.84	1.21	PREDICTED: transcription repressor MYB4 [*Eucalyptus grandis*]
miR8712	9.89	-0.83	0.23	-1.77	-0.19	1.52	8.96	9.37	0.38	*Gglean024246.1*	0.00	1.51	0.00	3.35	0.00	-1.62	0.00	0.00	0.00	retrotransposon-like protein [*Musa acuminata*]
miR8712	9.89	-0.83	0.23	-1.77	-0.19	1.52	8.96	9.37	0.38	*Gglean025906.1*	0.11	0.62	1.15	0.50	1.92	1.43	0.00	0.88	0.91	EXS family protein [*Theobroma cacao*]

miR8712	9.89	-0.83	0.23	-1.77	-0.19	1.52	8.96	9.37	0.38	*Gglean009062.1*	-0.33	1.54	1.48	2.87	2.99	0.13	1.01	1.17	0.19	PREDICTED: sugar transport protein 14 [*Eucalyptus grandis*]
miR902j-5p	NA[b]	-0.72	13.82	NA[b]	13.76	1.03	13.33	NA[b]	-13.55	*Gglean020069.1*	0.00	0.53	0.23	0.68	0.54	-0.13	0.16	0.30	0.17	PREDICTED: ankyrin repeat and SAM domain-containing protein 6-like [*Eucalyptus grandis*]
miR902j-5p	NA[b]	-0.72	13.82	NA[b]	13.76	1.03	13.33	NA[b]	-13.55	*Gglean017067.1*	-0.15	0.83	0.58	1.31	1.75	0.45	0.34	1.02	0.70	hypothetical protein PRUPE_ppa018570mg, partial [*Prunus persica*]
miR902j-5p	NA[b]	-0.72	13.82	NA[b]	13.76	1.03	13.33	NA[b]	-13.55	*Gglean009864.1*	-0.37	-1.10	-0.09	-1.32	2.43	3.76	-0.57	2.15	2.75	PREDICTED: uncharacterized protein LOC104418924 [*Eucalyptus grandis*]
miR952b	-1.85	-0.09	14.53	1.64	2.76	1.20	-0.10	-13.72	-13.63	*Gglean017511.1*	-0.04	0.63	0.74	0.49	0.76	0.27	-0.16	-0.03	0.16	PREDICTED: presequence protease 2, chloroplastic/mitochondrial-like [*Eucalyptus grandis*]
miR952b	-1.85	-0.09	14.53	1.64	2.76	1.20	-0.10	-13.72	-13.63	*Gglean014820.1*	-0.35	-1.33	-0.14	-0.77	-0.06	0.72	0.23	-0.27	-0.47	PREDICTED: jacalin-related lectin 4-like isoform X2 [*Eucalyptus grandis*]
miR952b	-1.85	-0.09	14.53	1.64	2.76	1.20	-0.10	-13.72	-13.63	*Gglean010890.1*	-0.10	0.47	0.24	0.54	0.63	0.10	-0.02	0.28	0.33	PREDICTED: protein PLANT CADMIUM RESISTANCE 10 [*Eucalyptus grandis*]
miR952b	-1.85	-0.09	14.53	1.64	2.76	1.20	-0.10	-13.72	-13.63	*Gglean001502.1*	-0.48	0.47	1.01	1.61	2.31	0.71	0.67	0.81	0.17	PREDICTED: uncharacterized protein LOC104445657 [*Eucalyptus grandis*]
miR952b	-1.85	-0.09	14.53	1.64	2.76	1.20	-0.10	-13.72	-13.63	*Gglean018134.1*	0.03	-2.62	-2.38	-3.18	-3.52	-0.34	-0.51	-1.12	-0.59	PREDICTED: uncharacterized protein LOC104454517 isoform X2 [*Eucalyptus grandis*]
miR952b	-1.85	-0.09	14.53	1.64	2.76	1.20	-0.10	-13.72	-13.63	*Gglean002174.1*	0.10	0.62	0.65	0.36	-0.06	-0.41	-0.15	-0.61	-0.43	PREDICTED: aspartate aminotransferase, cytoplasmic-like isoform X1 [*Eucalyptus grandis*]
miR952b	-1.85	-0.09	14.53	1.64	2.76	1.20	-0.10	-13.72	-13.63	*Gglean014819.1*	-0.06	0.17	1.28	0.50	1.73	1.25	0.27	0.39	0.14	PREDICTED: jacalin-related lectin 4-like isoform X2 [*Eucalyptus grandis*]
miR952b	-1.85	-0.09	14.53	1.64	2.76	1.20	-0.10	-13.72	-13.63	*Gglean026783.1*	0.00	2.28	1.33	4.68	4.83	0.16	0.00	0.00	0.00	hypothetical protein CICLE_v10031321mg [*Citrus clementina*]
miR408-3p	0.26	3.02	0.73	2.82	0.72	-2.04	0.11	0.15	0.01	*Gglean029859.1*	-0.31	-0.83	-2.69	-0.83	-2.28	-1.44	-0.29	0.09	0.40	Basic blue protein [*Medicago truncatula*]

miR408-3p	0.26	3.02	0.73	2.82	0.72	-2.04	0.11	0.15	0.01	*Gglean030438.1*	0.00	-0.99	-1.19	0.00	0.00	0.00	0.48	0.51	0.07	Laccase 3 [*Theobroma cacao*]
miR408-3p	0.26	3.02	0.73	2.82	0.72	-2.04	0.11	0.15	0.01	*Gglean022081.1*	0.13	0.51	0.44	0.56	0.56	0.01	0.18	0.24	0.08	PREDICTED: zinc finger with UFM1-specific peptidase domain protein [*Eucalyptus grandis*]
miR408-3p	0.26	3.02	0.73	2.82	0.72	-2.04	0.11	0.15	0.01	*Gglean027383.1*	0.06	0.75	-0.17	1.05	-0.27	-1.31	0.38	-0.04	-0.39	PREDICTED: callose synthase 7 [*Prunus mume*]
miR408-3p	0.26	3.02	0.73	2.82	0.72	-2.04	0.11	0.15	0.01	*Gglean010813.1*	0.09	-0.45	-0.43	-0.66	-0.56	0.11	-0.11	-0.05	0.08	PREDICTED: pentatricopeptide repeat-containing protein At3g58590 [*Eucalyptus grandis*]
miR7782-3p	-1.00	-0.07	0.47	1.41	1.78	0.40	0.48	0.22	-0.27	*Gglean027463.1*	0.00	0.00	0.00	0.00	0.00	0.00	0.00	0.00	0.00	PREDICTED: defensin-like protein 182 [*Malus domestica*]
miR5152-3p	-0.07	0.29	9.71	0.49	1.05	0.58	0.05	-8.88	-8.94	*Gglean006196.1*	0.05	0.63	0.40	0.50	0.61	0.12	-0.07	0.25	0.35	aspartyl protease family protein [*Populus trichocarpa*]
miR5223	-0.43	-0.32	-9.10	-0.07	-8.93	-8.82	-0.16	-0.34	-0.22	*Gglean001412.1*	0.08	-0.06	0.46	-0.11	-0.68	-0.56	0.04	-1.07	-1.08	hypothetical protein JCGZ_07785 [*Jatropha curcas*]
miR171b	-0.44	-0.05	-0.56	0.09	-0.45	-0.51	-0.30	-0.42	-0.15	*Gglean025172.1*	-0.18	0.99	0.87	1.97	2.42	0.46	0.81	1.37	0.59	PREDICTED: scarecrow-like protein 15 [*Eucalyptus grandis*]
miR171b	-0.44	-0.05	-0.56	0.09	-0.45	-0.51	-0.30	-0.42	-0.15	*Gglean006729.1*	-0.19	0.86	-0.15	1.11	-0.78	-1.88	0.07	-0.82	-0.86	PREDICTED: scarecrow-like protein 6 [*Eucalyptus grandis*]
miR171b-3p	0.54	-0.22	-0.03	-0.69	-0.79	-0.08	0.04	-0.34	-0.41	*Gglean025172.1*	-0.18	0.99	0.87	1.97	2.42	0.46	0.81	1.37	0.59	PREDICTED: scarecrow-like protein 15 [*Eucalyptus grandis*]
miR171b-3p	0.54	-0.22	-0.03	-0.69	-0.79	-0.08	0.04	-0.34	-0.41	*Gglean006729.1*	-0.19	0.86	-0.15	1.11	-0.78	-1.88	0.07	-0.82	-0.86	PREDICTED: scarecrow-like protein 6 [*Eucalyptus grandis*]
miR8712	9.89	-0.83	0.23	-1.77	-0.19	1.52	8.96	9.37	0.38	*Gglean026247.1*	0.00	0.00	0.00	0.00	0.00	0.00	0.00	0.00	0.00	unnamed protein product [*Nicotiana tabacum*]
miR8712	9.89	-0.83	0.23	-1.77	-0.19	1.52	8.96	9.37	0.38	*Gglean015249.1*	0.00	0.00	0.00	0.00	0.00	0.00	0.00	0.00	0.00	unnamed protein product [*Nicotiana tabacum*]

miR8712	9.89	-0.83	0.23	-1.77	-0.19	1.52	8.96	9.37	0.38	*Gglean004616.1*	0.00	0.00	0.00	0.00	0.00	0.00	0.00	0.00	0.00	unnamed protein product [*Nicotiana tabacum*]
miR8712	9.89	-0.83	0.23	-1.77	-0.19	1.52	8.96	9.37	0.38	*Gglean024246.1*	0.00	1.51	0.00	3.35	0.00	-1.62	0.00	0.00	0.00	retrotransposon-like protein [*Musa acuminata*]
miR8712	9.89	-0.83	0.23	-1.77	-0.19	1.52	8.96	9.37	0.38	*Gglean026959.1*	0.00	0.00	0.00	0.00	0.00	0.00	0.00	0.00	0.00	unnamed protein product [*Nicotiana tabacum*]
miR8712	9.89	-0.83	0.23	-1.77	-0.19	1.52	8.96	9.37	0.38	*Gglean008852.1*	0.00	0.00	0.00	0.00	0.00	0.00	0.00	0.00	0.00	unnamed protein product [*Nicotiana tabacum*]
miR8712	9.89	-0.83	0.23	-1.77	-0.19	1.52	8.96	9.37	0.38	*Gglean019316.1*	0.00	0.00	0.00	0.00	0.00	0.00	0.00	0.00	0.00	hypothetical protein VITISV_007384 [*Vitis vinifera*]
miR8712	9.89	-0.83	0.23	-1.77	-0.19	1.52	8.96	9.37	0.38	*Gglean025906.1*	0.11	0.62	1.15	0.50	1.92	1.43	0.00	0.88	0.91	EXS family protein [*Theobroma cacao*]
miR8712	9.89	-0.83	0.23	-1.77	-0.19	1.52	8.96	9.37	0.38	*Gglean009062.1*	-0.33	1.54	1.48	2.87	2.99	0.13	1.01	1.17	0.19	PREDICTED: sugar transport protein 14 [*Eucalyptus grandis*]
miR8712	9.89	-0.83	0.23	-1.77	-0.19	1.52	8.96	9.37	0.38	*Gglean015308.1*	0.00	0.00	0.00	0.00	0.00	0.00	0.00	0.00	0.00	unnamed protein product [*Nicotiana tabacum*]
miR8712	9.89	-0.83	0.23	-1.77	-0.19	1.52	8.96	9.37	0.38	*Gglean006955.1*	0.00	0.00	0.00	0.00	0.00	0.00	0.00	0.00	0.00	hypothetical retrotransposon [*Ipomoea batatas*]
miR6300	-2.89	1.65	-0.33	1.28	1.09	-0.14	-3.29	-1.63	1.67	*Gglean009658.1*	-0.02	0.34	-0.04	0.85	0.62	-0.23	0.51	0.63	0.16	PREDICTED: probable polyamine transporter At1g31830 isoform X2 [*Eucalyptus grandis*]
miR6300	-2.89	1.65	-0.33	1.28	1.09	-0.14	-3.29	-1.63	1.67	*Gglean008406.1*	0.02	-0.16	0.13	-0.45	-0.08	0.38	-0.26	-0.19	0.09	PREDICTED: putative pentatricopeptide repeat-containing protein At5g08310, mitochondrial [*Eucalyptus grandis*]
miR6300	-2.89	1.65	-0.33	1.28	1.09	-0.14	-3.29	-1.63	1.67	*Gglean022739.1*	0.22	0.33	0.31	0.14	0.09	-0.04	0.05	0.00	-0.02	PREDICTED: OBERON-like protein [*Populus euphratica*]
miR6300	-2.89	1.65	-0.33	1.28	1.09	-0.14	-3.29	-1.63	1.67	*Gglean013458.1*	0.00	1.93	1.68	3.85	4.59	0.76	0.00	0.00	0.00	myb family transcription factor family protein [*Populus trichocarpa*]

miR6300	-2.89	1.65	-0.33	1.28	1.09	-0.14	-3.29	-1.63	1.67	*Gglean016906.1*	-0.17	0.34	-0.09	0.22	0.16	-0.05	-0.28	0.07	0.39	PREDICTED: peroxisome biogenesis protein 7 [*Eucalyptus grandis*]
miR6300	-2.89	1.65	-0.33	1.28	1.09	-0.14	-3.29	-1.63	1.67	*Gglean018041.1*	0.44	1.71	3.43	0.31	-0.15	-0.45	-0.94	-3.14	-2.16	PREDICTED: regulatory protein NPR5 [*Vitis vinifera*]
miR390a-5p	-0.12	-0.16	-0.20	-0.70	-1.03	-0.30	-0.67	-1.05	-0.41	*Gglean012251.1*	0.00	0.00	0.00	0.00	0.00	0.00	0.00	0.00	0.00	Leucine-rich repeat receptor protein kinase EXS precursor, putative [*Ricinus communis*]
miR390a-5p	-0.12	-0.16	-0.20	-0.70	-1.03	-0.30	-0.67	-1.05	-0.41	*Gglean025452.1*	-0.31	-0.44	0.29	-0.26	0.10	0.37	-0.12	-0.51	-0.36	PREDICTED: uncharacterized protein LOC101315171 [*Fragaria vesca* subsp. vesca]
miR390a-5p	-0.12	-0.16	-0.20	-0.70	-1.03	-0.30	-0.67	-1.05	-0.41	*Gglean022261.1*	0.00	0.00	0.00	0.00	0.00	0.00	0.00	0.00	0.00	hypothetical protein EUGRSUZ_F02430 [*Eucalyptus grandis*]
miR390a-5p	-0.12	-0.16	-0.20	-0.70	-1.03	-0.30	-0.67	-1.05	-0.41	*Gglean000925.1*	-0.27	-0.27	-0.10	-0.09	0.03	0.12	-0.07	-0.16	-0.06	PREDICTED: dnaJ homolog subfamily B member 1 [*Eucalyptus grandis*]
miR390a-5p	-0.12	-0.16	-0.20	-0.70	-1.03	-0.30	-0.67	-1.05	-0.41	*Gglean003665.1*	-0.37	0.57	0.47	0.83	0.99	0.17	-0.09	0.15	0.27	PREDICTED: serine/threonine-protein kinase BRI1-like 1 [*Populus euphratica*]
miR396b-5p	0.40	0.11	-0.30	0.68	0.95	0.32	1.00	1.58	0.54	*Gglean029554.1*	0.00	0.00	0.00	0.00	0.00	0.00	0.00	0.00	0.00	PREDICTED: putative B3 domain-containing protein At3g24850 [*Vitis vinifera*]
miR396b-5p	0.40	0.11	-0.30	0.68	0.95	0.32	1.00	1.58	0.54	*Gglean002260.1*	0.26	0.15	-0.51	-0.61	-2.17	-1.55	-0.49	-1.41	-0.90	PREDICTED: small glutamine-rich tetratricopeptide repeat-containing protein 2 [*Tarenaya hassleriana*]
miR396b-5p	0.40	0.11	-0.30	0.68	0.95	0.32	1.00	1.58	0.54	*Gglean026508.1*	-0.06	-0.53	-0.39	-0.48	-0.50	-0.01	0.00	-0.17	-0.14	hypothetical protein JCGZ_06261 [*Jatropha curcas*]
miR396b-5p	0.40	0.11	-0.30	0.68	0.95	0.32	1.00	1.58	0.54	*Gglean011692.1*	-0.19	-0.28	0.10	-0.12	0.13	0.26	-0.02	-0.17	-0.12	hypothetical protein EUGRSUZ_D00484 [*Eucalyptus grandis*]
miR396b-5p	0.40	0.11	-0.30	0.68	0.95	0.32	1.00	1.58	0.54	*Gglean017067.1*	-0.15	0.83	0.58	1.31	1.75	0.45	0.34	1.02	0.70	hypothetical protein PRUPE_ppa018570mg, partial [*Prunus persica*]
miR396b-5p	0.40	0.11	-0.30	0.68	0.95	0.32	1.00	1.58	0.54	*Gglean012701.1*	0.00	0.00	0.00	0.00	0.00	0.00	0.00	0.00	0.00	PREDICTED: uncharacterized protein LOC104417748 [*Eucalyptus grandis*]

miR827-5p	-0.37	0.98	0.34	-0.80	-0.88	-0.03	-2.13	-1.69	0.41	*Gglean013516.1*	-0.28	0.17	0.36	0.91	1.51	0.61	0.48	0.86	0.42	Duplicated homeodomain-like superfamily protein, putative isoform 1 [*Theobroma cacao*]
miR827-5p	-0.37	0.98	0.34	-0.80	-0.88	-0.03	-2.13	-1.69	0.41	*Gglean022904.1*	0.00	0.00	0.00	0.00	0.00	0.00	0.00	0.00	0.00	PREDICTED: transcription initiation factor TFIID subunit 1 isoform X1 [*Eucalyptus grandis*]
miR827-5p	-0.37	0.98	0.34	-0.80	-0.88	-0.03	-2.13	-1.69	0.41	*Gglean006537.1*	0.00	0.00	-6.07	0.00	0.00	0.00	0.00	12.27	5.31	PREDICTED: MADS-box protein FBP24-like [*Eucalyptus grandis*]
miR319a-3p	-0.34	-0.17	0.78	-1.78	-2.20	-0.39	-1.98	-3.42	-1.48	*Gglean024699.1*	0.00	0.00	0.00	0.00	0.00	0.00	0.00	0.00	0.00	NA[b]
miR319a-3p	-0.34	-0.17	0.78	-1.78	-2.20	-0.39	-1.98	-3.42	-1.48	*Gglean017814.1*	-0.24	0.36	-0.02	1.14	0.48	-0.65	0.55	0.26	-0.27	PREDICTED: transcription factor GAMYB [*Eucalyptus grandis*]
miR319a-3p	-0.34	-0.17	0.78	-1.78	-2.20	-0.39	-1.98	-3.42	-1.48	*Gglean016054.1*	0.37	-0.99	-1.01	-1.79	-2.04	-0.24	-0.41	-0.67	-0.23	PREDICTED: transcription factor GAMYB [*Eucalyptus grandis*]
miR319a-3p	-0.34	-0.17	0.78	-1.78	-2.20	-0.39	-1.98	-3.42	-1.48	*Gglean022391.1*	0.16	-1.81	-1.86	-2.78	-3.66	-0.88	-0.79	-1.65	-0.83	PREDICTED: kinesin-like protein KIF22-A [*Prunus mume*]
miR319a-3p	-0.34	-0.17	0.78	-1.78	-2.20	-0.39	-1.98	-3.42	-1.48	*Gglean028556.1*	0.05	-0.12	-0.45	-0.28	-0.50	-0.21	-0.10	-0.01	0.12	Protein FRIGIDA -like protein [*Gossypium arboreum*]
miR319a-3p	-0.34	-0.17	0.78	-1.78	-2.20	-0.39	-1.98	-3.42	-1.48	*Gglean013014.1*	-0.25	0.96	0.37	1.80	1.36	-0.43	0.61	0.74	0.16	Uncharacterized protein F383_17343 [*Gossypium arboreum*]
miR319a-3p	-0.34	-0.17	0.78	-1.78	-2.20	-0.39	-1.98	-3.42	-1.48	*Gglean000711.1*	0.12	-0.49	-0.44	-0.72	-1.31	-0.58	-0.10	-0.76	-0.64	PREDICTED: A/G-specific adenine DNA glycosylase isoform X1 [*Eucalyptus grandis*]
miRN01	1.43	-2.83	1.65	-2.76	-0.67	NA[b]	1.60	NA[b]	-2.67	*Gglean031077.1*	0.00	0.00	0.00	0.00	0.00	0.00	0.00	0.00	0.00	PREDICTED: zinc finger protein ZAT9-like [*Nicotiana tomentosiformis*]
miRN01	1.43	-2.83	1.65	-2.76	-0.67	NA[b]	1.60	NA[b]	-2.67	*Gglean020871.1*	0.00	0.00	0.00	0.00	0.00	0.00	0.00	0.00	0.00	PREDICTED: anthocyanin regulatory C1 protein-like [*Eucalyptus grandis*]
miRN01	1.43	-2.83	1.65	-2.76	-0.67	NA[b]	1.60	NA[b]	-2.67	*Gglean023939.1*	0.00	2.16	0.06	3.34	4.36	1.03	1.08	4.18	3.12	PREDICTED: myb-related protein Zm38-like [*Prunus mume*]

miRN01	1.43	-2.83	1.65	-2.76	-0.67	NA[b]	1.60	NA[b]	-2.67	*Gglean004276.1*	0.22	-0.79	-3.64	-0.83	-1.16	-0.32	0.18	2.69	2.53	PREDICTED: transcription factor WER-like [*Eucalyptus grandis*]
miRN01	1.43	-2.83	1.65	-2.76	-0.67	NA[b]	1.60	NA[b]	-2.67	*Gglean027607.1*	-0.52	-0.59	0.13	0.18	1.26	1.09	0.27	0.60	0.36	PREDICTED: transcription factor MYB12-like [*Glycine max*]
miRN01	1.43	-2.83	1.65	-2.76	-0.67	NA[b]	1.60	NA[b]	-2.67	*Gglean008242.1*	3.10	2.75	3.18	2.96	1.83	-1.11	0.00	0.00	-1.54	myb-related transcription factor [*Panax ginseng*]
miRN01	1.43	-2.83	1.65	-2.76	-0.67	NA[b]	1.60	NA[b]	-2.67	*Gglean012452.1*	-2.68	-1.71	-2.92	3.61	3.60	0.00	2.65	3.84	1.21	PREDICTED: transcription repressor MYB4 [*Eucalyptus grandis*]
miRN01	1.43	-2.83	1.65	-2.76	-0.67	NA[b]	1.60	NA[b]	-2.67	*Gglean004315.1*	-0.15	2.77	1.00	7.29	4.16	-3.12	4.39	3.02	-1.35	Duplicated homeodomain-like superfamily protein [*Theobroma cacao*]
miRN30	1.12	-10.55	-9.58	-9.87	-9.89	NA[b]	1.84	0.76	-1.15	*Gglean016215.1*	-0.05	-1.05	-0.19	-1.27	-0.94	0.34	-0.27	-0.81	-0.52	60S ribosomal protein L17-2 [*Morus notabilis*]
miRN30	1.12	-10.55	-9.58	-9.87	-9.89	NA[b]	1.84	0.76	-1.15	*Gglean017324.1*	-0.20	0.35	0.14	0.32	-0.04	-0.35	-0.21	-0.39	-0.15	ICE transcription factor 1 [*Eucalyptus globulus*]
miRN30	1.12	-10.55	-9.58	-9.87	-9.89	NA[b]	1.84	0.76	-1.15	*Gglean011392.1*	-0.20	0.20	0.40	0.41	0.94	0.55	0.02	0.34	0.34	PREDICTED: pentatricopeptide repeat-containing protein At4g25270, chloroplastic [*Eucalyptus grandis*]
miRN31	-0.77	9.60	-2.02	-0.08	-1.00	-0.91	-10.41	0.17	10.54	*Gglean004741.1*	-0.11	-0.01	-0.20	-0.16	-2.65	-2.48	-0.25	-2.57	-2.31	unnamed protein product [*Vitis vinifera*]
miRN31	-0.77	9.60	-2.02	-0.08	-1.00	-0.91	-10.41	0.17	10.54	*Gglean028306.1*	-0.02	-0.05	-0.17	0.25	-1.35	-1.60	0.30	-1.21	-1.48	LIGULELESS1 protein, putative [*Theobroma cacao*]
miRN31	-0.77	9.60	-2.02	-0.08	-1.00	-0.91	-10.41	0.17	10.54	*Gglean012943.1*	-0.07	0.58	0.26	0.96	0.57	-0.38	0.31	0.22	-0.06	unnamed protein product [*Vitis vinifera*]
miRN31	-0.77	9.60	-2.02	-0.08	-1.00	-0.91	-10.41	0.17	10.54	*Gglean028488.1*	0.16	-0.78	-0.80	-1.69	-2.69	-0.99	-0.74	-1.75	-0.98	PREDICTED: squamosa promoter-binding-like protein 13A [*Vitis vinifera*]
miRN31	-0.77	9.60	-2.02	-0.08	-1.00	-0.91	-10.41	0.17	10.54	*Gglean016208.1*	-0.07	0.49	0.27	0.00	0.26	-0.42	0.14	-0.09	-0.20	PREDICTED: squamosa promoter-binding-like protein 2 [*Eucalyptus grandis*]
miRN31	-0.77	9.60	-2.02	-0.08	-1.00	-0.91	-10.41	0.17	10.54	*Gglean000847.1*	-0.14	0.32	-0.05	0.14	-0.36	-0.49	-0.31	-0.46	-0.12	LIGULELESS1 protein, putative [*Theobroma cacao*]
miRN31	-0.77	9.60	-2.02	-0.08	-1.00	-0.91	-10.41	0.17	10.54	*Gglean016197.1*	-0.20	-0.08	-0.84	0.06	-2.10	-2.15	-0.04	-1.46	-1.40	PREDICTED: squamosa promoter-binding-like protein 16 [*Vitis vinifera*]

miRN07	0.64	-13.21	1.35	-14.10	-0.02	14.12	-0.20	-0.76	-0.64	*Gglean013966.1*	0.10	-1.19	-1.22	-2.15	-3.57	-1.41	-0.85	-2.26	-1.39	PREDICTED: homeobox-leucine zipper protein ATHB-8-like [*Eucalyptus grandis*]
miRN07	0.64	-13.21	1.35	-14.10	-0.02	14.12	-0.20	-0.76	-0.64	*Gglean027183.1*	0.07	-0.77	-0.33	-1.29	-1.73	-0.42	-0.45	-1.34	-0.86	HB1-like protein [*Eucalyptus cladocalyx*]
miRN07	0.64	-13.21	1.35	-14.10	-0.02	14.12	-0.20	-0.76	-0.64	*Gglean010679.1*	-0.07	-1.01	-0.38	-1.24	-0.98	0.27	-0.28	-0.67	-0.36	PREDICTED: 60S ribosomal protein L9 [*Eucalyptus grandis*]
miRN07	0.64	-13.21	1.35	-14.10	-0.02	14.12	-0.20	-0.76	-0.64	*Gglean031286.1*	-0.14	-0.42	-0.76	-0.32	-1.26	-0.93	-0.03	-0.66	-0.60	DNA binding protein, putative [*Ricinus communis*]
miRN48	-0.36	1.96	-7.52	0.65	-6.92	-21.04	-1.57	0.14	1.66	*Gglean011726.1*	0.00	0.00	0.00	0.00	0.00	0.00	0.00	0.00	0.00	hypothetical protein VOLCADRAFT_98215 [*Volvox carteri f. nagariensis*]
miRN48	-0.36	1.96	-7.52	0.65	-6.92	-21.04	-1.57	0.14	1.66	*Gglean011729.1*	0.00	0.00	0.00	0.00	0.00	0.00	0.00	0.00	0.00	hypothetical protein VOLCADRAFT_98215 [*Volvox carteri f. nagariensis*]
miRN11	0.90	-13.15	-0.28	-13.88	-3.71	NA[b]	0.18	-2.63	-2.85	*Gglean007369.1*	-0.29	1.68	2.16	2.08	7.74	5.67	0.12	5.30	5.21	PREDICTED: nodulation-signaling pathway 2 protein-like [*Citrus sinensis*]
miRN11	0.90	-13.15	-0.28	-13.88	-3.71	NA[b]	0.18	-2.63	-2.85	*Gglean006729.1*	-0.19	0.86	-0.15	1.11	-0.78	-1.88	0.07	-0.82	-0.86	PREDICTED: scarecrow-like protein 6 [*Eucalyptus grandis*]
miRN11	0.90	-13.15	-0.28	-13.88	-3.71	NA[b]	0.18	-2.63	-2.85	*Gglean026987.1*	0.24	-2.07	0.51	-2.40	-1.27	1.13	-0.08	-1.55	-1.45	PREDICTED: acid beta-fructofuranosidase-like [*Eucalyptus grandis*]
miRN11	0.90	-13.15	-0.28	-13.88	-3.71	NA[b]	0.18	-2.63	-2.85	*Gglean003203.1*	0.00	0.00	0.00	0.00	0.00	0.00	0.00	0.00	0.00	PREDICTED: probably inactive leucine-rich repeat receptor-like protein kinase At3g28040 [*Eucalyptus grandis*]
miRN11	0.90	-13.15	-0.28	-13.88	-3.71	NA[b]	0.18	-2.63	-2.85	*Gglean024398.1*	0.42	0.54	-0.27	-0.42	-2.31	-1.88	-0.53	-1.62	-1.07	hypothetical protein CICLE_v10006513mg [*Citrus clementina*]
miRN11	0.90	-13.15	-0.28	-13.88	-3.71	NA[b]	0.18	-2.63	-2.85	*Gglean025172.1*	-0.18	0.99	0.87	1.97	2.42	0.46	0.81	1.37	0.59	PREDICTED: scarecrow-like protein 15 [*Eucalyptus grandis*]
miRN11	0.90	-13.15	-0.28	-13.88	-3.71	NA[b]	0.18	-2.63	-2.85	*Gglean006444.1*	-0.12	0.46	0.35	0.90	0.99	0.10	0.33	0.52	0.22	Phosphoenolpyruvate carboxylase, putative [*Ricinus communis*]

miRN11	0.90	-13.15	-0.28	-13.88	-3.71	NA[b]	0.18	-2.63	-2.85	*Gglean025172.1*	-0.18	0.99	0.87	1.97	2.42	0.46	0.81	1.37	0.59	PREDICTED: scarecrow-like protein 15 [*Eucalyptus grandis*]
miRN11	0.90	-13.15	-0.28	-13.88	-3.71	NA[b]	0.18	-2.63	-2.85	*Gglean027349.1*	0.69	0.64	0.56	-0.80	-0.05	0.76	-0.73	0.08	0.85	PREDICTED: uncharacterized protein LOC105170581 [*Sesamum indicum*]
miRN11	0.90	-13.15	-0.28	-13.88	-3.71	NA[b]	0.18	-2.63	-2.85	*Gglean014673.1*	0.45	1.78	1.58	2.76	2.18	-0.57	1.45	1.06	-0.36	PREDICTED: SNF1-related protein kinase regulatory subunit beta-1 [*Vitis vinifera*]
miRN11	0.90	-13.15	-0.28	-13.88	-3.71	NA[b]	0.18	-2.63	-2.85	*Gglean004494.1*	-0.04	0.27	0.43	0.33	0.55	0.23	0.04	0.09	0.08	PREDICTED: mitochondrial ribosome-associated GTPase 1 isoform X1 [*Eucalyptus grandis*]
miRN11	0.90	-13.15	-0.28	-13.88	-3.71	NA[b]	0.18	-2.63	-2.85	*Gglean006729.1*	-0.19	0.86	-0.15	1.11	-0.78	-1.88	0.07	-0.82	-0.86	PREDICTED: scarecrow-like protein 6 [*Eucalyptus grandis*]
miRN56	-11.61	1.86	-0.97	13.34	22.49	-2.80	-0.02	-0.26	-0.30	*Gglean007834.1*	0.00	0.00	0.00	0.00	0.00	0.00	0.00	0.00	0.00	PREDICTED: Prunus mume O-glucosyltransferase rumi homolog (LOC103331895), mRNA
miRN56	-11.61	1.86	-0.97	13.34	22.49	-2.80	-0.02	-0.26	-0.30	*Gglean003381.1*	0.00	5.77	0.82	7.06	4.10	-2.95	0.00	0.00	1.99	PREDICTED: Pyrus x bretschneideri NAC domain-containing protein 21/22-like (LOC103929240), mRNA
miRN56	-11.61	1.86	-0.97	13.34	22.49	-2.80	-0.02	-0.26	-0.30	*Gglean003444.1*	0.45	0.11	-0.55	-1.72	-5.76	-4.03	-1.37	3.91	-3.38	PREDICTED: *Eucalyptus grandis* protein CUP-SHAPED COTYLEDON 2 (LOC104451522), mRNA
miRN56	-11.61	1.86	-0.97	13.34	22.49	-2.80	-0.02	-0.26	-0.30	*Gglean026964.1*	-0.11	-0.41	-2.03	-0.12	0.88	1.01	0.20	-4.77	2.64	PREDICTED: *Populus euphratica* UDP-glucuronic acid decarboxylase 6-like (LOC105127148), transcript variant X2, mRNA
miRN56	-11.61	1.86	-0.97	13.34	22.49	-2.80	-0.02	-0.26	-0.30	*Gglean022642.1*	0.00	0.00	0.00	0.00	0.00	0.00	0.00	0.00	0.00	PREDICTED: *Elaeis guineensis* thaumatin-like protein 1b (LOC105053748), mRNA

miRN36	1.87	-9.31	-10.48	-8.36	-8.37	NA[b]	2.90	3.95	1.02	*Gglean003827.1*	0.04	-0.13	0.20	-0.11	0.79	0.91	0.06	0.62	0.59	Populus trichocarpa WD-40 repeat family protein (POPTR_0011s12320g) mRNA, complete cds
miRN36	1.87	-9.31	-10.48	-8.36	-8.37	NA[b]	2.90	3.95	1.02	*Gglean007369.1*	-0.29	1.68	2.16	2.08	7.74	5.67	0.12	5.30	5.21	PREDICTED: nodulation-signaling pathway 2 protein-like [*Citrus sinensis*]
miRN36	1.87	-9.31	-10.48	-8.36	-8.37	NA[b]	2.90	3.95	1.02	*Gglean029630.1*	-0.03	-2.73	-2.69	-3.77	-4.14	-0.36	-1.06	-1.49	-0.41	PREDICTED: Eucalyptus grandis BRCT domain-containing protein At4g02110 (LOC104426812), mRNA
miRN38	0.16	0.18	0.91	1.22	2.26	1.08	1.20	1.39	0.17	*Gglean017109.1*	0.03	0.28	0.07	0.07	-0.35	-0.41	-0.17	-0.39	-0.20	auxin signaling f-box 2 -like protein [*Gossypium arboreum*]
miRN38	0.16	0.18	0.91	1.22	2.26	1.08	1.20	1.39	0.17	*Gglean006519.1*	-0.15	0.26	-0.27	0.36	-0.33	-0.68	-0.03	-0.22	-0.16	PREDICTED: protein TRANSPORT INHIBITOR RESPONSE 1 [*Vitis vinifera*]
miRN13	1.52	-0.69	-12.69	-1.76	-14.31	-12.46	0.42	-0.23	-0.63	*Gglean001744.1*	0.00	0.00	0.00	0.00	0.00	0.00	0.00	0.00	0.00	NA[b]
miRN18	-0.63	21.48	NA[b]	-4.47	-12.50	-21.47	-13.18	-13.20	NA[b]	*Gglean017814.1*	-0.24	0.36	-0.02	1.14	0.48	-0.65	0.55	0.26	-0.27	PREDICTED: transcription factor GAMYB [*Eucalyptus grandis*]
miRN18	-0.63	21.48	NA[b]	-4.47	-12.50	-21.47	-13.18	-13.20	NA[b]	*Gglean013014.1*	-0.25	0.96	0.37	1.80	1.36	-0.43	0.61	0.74	0.16	Uncharacterized protein F383_17343 [*Gossypium arboreum*]
miRN18	-0.63	21.48	NA[b]	-4.47	-12.50	-21.47	-13.18	-13.20	NA[b]	*Gglean015155.1*	0.34	0.88	0.63	0.76	0.68	-0.08	0.23	0.38	0.17	PREDICTED: protein SENSITIVE TO PROTON RHIZOTOXICITY 1 [*Eucalyptus grandis*]
miRN18	-0.63	21.48	NA[b]	-4.47	-12.50	-21.47	-13.18	-13.20	NA[b]	*Gglean013127.1*	-0.27	0.32	-0.43	0.97	0.36	-0.61	0.40	0.51	0.14	hypothetical protein PRUPE_ppa004612mg [*Prunus persica*]
miRN18	-0.63	21.48	NA[b]	-4.47	-12.50	-21.47	-13.18	-13.20	NA[b]	*Gglean006071.1*	0.00	0.00	0.00	0.00	0.00	0.00	0.00	0.00	0.00	PREDICTED: uncharacterized protein LOC104450247 [*Eucalyptus grandis*]
miRN18	-0.63	21.48	NA[b]	-4.47	-12.50	-21.47	-13.18	-13.20	NA[b]	*Gglean016054.1*	0.37	-0.99	-1.01	-1.79	-2.04	-0.24	-0.41	-0.67	-0.23	PREDICTED: transcription factor GAMYB [*Eucalyptus grandis*]
miRN06	0.25	0.58	NA[b]	-0.82	-13.96	-13.13	NA[b]	NA[b]	-1.40	*Gglean020082.1*	-0.17	-1.42	-1.33	-4.21	-3.81	0.41	-2.94	-2.66	0.31	PREDICTED: uncharacterized protein LOC104417739 [*Eucalyptus grandis*]

miRN35	0.66	0.26	0.71	1.01	1.99	1.03	1.40	1.83	0.42	*Gglean020373.1*	0.00	0.00	0.00	0.00	0.00	0.00	0.00	0.00	0.00	NA[b]
miRN35	0.66	0.26	0.71	1.01	1.99	1.03	1.40	1.83	0.42	*Gglean010802.1*	0.14	0.27	0.02	0.28	0.42	0.15	0.16	0.53	0.40	RING/U-box superfamily protein isoform 1 [*Theobroma cacao*]
miRN35	0.66	0.26	0.71	1.01	1.99	1.03	1.40	1.83	0.42	*Gglean007104.1*	0.14	0.34	0.31	-0.45	-0.38	0.08	-0.64	-0.56	0.11	PREDICTED: NAC domain-containing protein 89-like [*Eucalyptus grandis*]
miRN12	2.54	-2.58	2.32	-2.69	-1.36	1.41	2.40	NA[b]	-3.71	*Gglean012868.1*	-0.05	-0.18	-0.26	-0.23	-0.51	-0.27	-0.09	-0.31	-0.19	PREDICTED: WD repeat-containing protein LWD1-like [*Cicer arietinum*]
miRN12	2.54	-2.58	2.32	-2.69	-1.36	1.41	2.40	NA[b]	-3.71	*Gglean010888.1*	0.03	0.39	0.18	0.70	0.39	-0.29	0.34	0.23	-0.08	PREDICTED: F-box protein SKIP24 [*Eucalyptus grandis*]
miRN12	2.54	-2.58	2.32	-2.69	-1.36	1.41	2.40	NA[b]	-3.71	*Gglean002551.1*	0.16	0.00	0.04	-0.27	-0.60	-0.32	-0.09	-0.49	-0.36	PREDICTED: pentatricopeptide repeat-containing protein At5g46580, chloroplastic [*Eucalyptus grandis*]
miRN12	2.54	-2.58	2.32	-2.69	-1.36	1.41	2.40	NA[b]	-3.71	*Gglean018747.1*	-0.05	0.78	0.95	1.32	1.30	-0.01	0.51	0.30	-0.18	PREDICTED: uncharacterized protein LOC103332870 [*Prunus mume*]

Note: b: values were filtered：-0.5＜log2fold change＜0.5 and adjusted P-value ＞0.05 or no annotation

Table S9 Primers used in this study

miRNA-ID	Stem loop reverse transcriptase primers	qRT-PCR upstream primers	qRT-PCR downstream universal primers
pg-miR166a-3p	GTCGTATCCAGTGCAGGGTCCGAGGTATTCGCACTGGATACGACGGGGAA	TCGGACCAGGCTTCATTCC	GTGCAGGGTCCGAGGT
pg-miR160a-3p	GTCGTATCCAGTGCAGGGTCCGAGGTATTCGCACTGGATACGACTATGCA	GCGTATGAGGAGCCATGCATA	GTGCAGGGTCCGAGGT
pg-miR167f-3p	GTCGTATCCAGTGCAGGGTCCGAGGTATTCGCACTGGATACGACGGTGAA	AGATCATGTGGTAGCTTCACC	GTGCAGGGTCCGAGGT
pg-miR397a	GTCGTATCCAGTGCAGGGTCCGAGGTATTCGCACTGGATACGACCATCAA	TGCTTCATTGAGTGCAGCG	GTGCAGGGTCCGAGGT
pg-miR398a	GTCGTATCCAGTGCAGGGTCCGAGGTATTCGCACTGGATACGACAAGGGG	GCCGTGTGTTTTCAGGTCA	GTGCAGGGTCCGAGGT
pg-miR398b	GTCGTATCCAGTGCAGGGTCCGAGGTATTCGCACTGGATACGACCAGGGG	ACTGCTGTGTTCTCAGGTCG	GTGCAGGGTCCGAGGT
pg-miR408-3p	GTCGTATCCAGTGCAGGGTCCGAGGTATTCGCACTGGATACGACGACCAG	ATGCACTGCCTCTTCCCTGG	GTGCAGGGTCCGAGGT
pg-miR4414b	GTCGTATCCAGTGCAGGGTCCGAGGTATTCGCACTGGATACGACGTATCT	AAGCTGTGAATGATGCGGG	GTGCAGGGTCCGAGGT
pg-miR5671a	GTCGTATCCAGTGCAGGGTCCGAGGTATTCGCACTGGATACGACGTCACC	TGCTGCATGGTGGTGACG	GTGCAGGGTCCGAGGT
pg-miR1514a-3p	GTCGTATCCAGTGCAGGGTCCGAGGTATTCGCACTGGATACGACTATTTC	CGGCGGATGCCTCGTTTTATAAT	GTGCAGGGTCCGAGGT

**miRNA-ID**	**Pre-miR166a sequence amplification**

pg-miR166a-3p-F	CGTCTAGAAACTCCCAACCCTAAAAGAACCCCTGTA
pg-miR166a-3p-R	ATCCCGGGAGAAATTGATTAGAGACTGAGA

**Target genes ID**	**primers for annealing**
pg-miR166a-3p-PC-F	GCCTCGAGTCGGGGAATGAAGCCTGGTCCGAGTCTAGACG
pg-miR166a-3p-PC-R	CGTCTAGACTCGGACCAGGCTTCATTCCCCGACTCGAGGC
Gglean013966.1-F	GCCTCGAGCTCTGGGATGAAGCCTGGTCCGGCTCTAGACG
Gglean013966.1-R	CGTCTAGAGCCGGACCAGGCTTCATCCCAGAGCTCGAGGC
Gglean012177.1-F	GCCTCGAGCCTTGGGATGAAGCCTGGTCCGGATCTAGACG
Gglean012177.1-R	CGTCTAGATCCGGACCAGGCTTCATCCCAAGGCTCGAGGC
M-F	GCCTCGAGCTGAGCGAGGATAGCAGACGGTCTAGACG
M-R	CGTCTAGACCGTCTGCTATCCTCGCTCAGCTCGAGGC
